# Variation in quality of acute stroke care by day and time of admission: prospective cohort study of weekday and weekend centralised hyperacute stroke unit care and non-centralised services

**DOI:** 10.1136/bmjopen-2018-025366

**Published:** 2019-11-07

**Authors:** Mariya Melnychuk, Stephen Morris, Georgia Black, Angus I G Ramsay, Jeannie Eng, Anthony Rudd, Abigail Baim-Lance, Martin M Brown, Naomi J Fulop, Robert Simister

**Affiliations:** 1 Department of Applied Health Research, University College London, London, UK; 2 Faculty of Law and Social Sciences, Universidad Rey Juan Carlos, Madrid, Spain; 3 Cancer Division, University College London Hospitals NHS Foundation Trust, London; 4 Clinical Effectiveness and Evaluation Unit, Royal College of Physicians, London, UK; 5 Institute for Implementation Science in Population Health, City University of New York, New York, USA; 6 Stroke Research Centre, UCL Queen Square Institute of Neurology, London; 7 Comprehensive Stroke Service, University College London Hospitals NHS Foundation Trust, London, UK

**Keywords:** health economics, stroke

## Abstract

**Objective:**

To investigate variations in quality of acute stroke care and outcomes by day and time of admission in London hyperacute stroke units compared with the rest of England.

**Design:**

Prospective cohort study using anonymised patient-level data from the Sentinel Stroke National Audit Programme.

**Setting:**

Acute stroke services in London hyperacute stroke units and the rest of England.

**Participants:**

68 239 patients with a primary diagnosis of stroke admitted between January and December 2014.

**Interventions:**

Hub-and-spoke model for care of suspected acute stroke patients in London with performance standards designed to deliver uniform access to high-quality hyperacute stroke unit care across the week.

**Main outcome measures:**

16 indicators of quality of acute stroke care, mortality at 3 days after admission to the hospital, disability at the end of the inpatient spell, length of stay.

**Results:**

There was no variation in quality of care by day and time of admission to the hospital across the week in terms of stroke nursing assessment, brain scanning and thrombolysis in London hyperacute stroke units, nor was there variation in 3-day mortality or disability at hospital discharge (all p values>0.05). Other quality of care measures significantly varied by day and time of admission across the week in London (all p values<0.01). In the rest of England there was variation in all measures by day and time of admission across the week (all p values<0.01), except for mortality at 3 days (p value>0.05).

**Conclusions:**

The London hyperacute stroke unit model achieved performance standards for ‘front door’ stroke care across the week. The same benefits were not achieved by other models of care in the rest of England. There was no weekend effect for mortality in London or the rest of the England. Other aspects of care were not constant across the week in London hyperacute stroke units, indicating some performance standards were perceived to be more important than others.

**Strengths and limitations of this study**

- We used a large national data set containing detailed information on quality of stroke care, outcomes and patient characteristics.
- We examined whether time of admission was related to quality of care using a comprehensive set of indicators from across the acute stroke care pathway to reflect the time-critical nature of acute stroke care.
- Our outcomes were stroke short-term mortality and disability, but we were unable to measure long-term outcomes as these were not available in Sentinel Stroke National Audit Programme.

## Introduction

There is conflicting evidence as to whether or not patients presenting with acute stroke symptoms receive lower quality of care and have worse outcomes if admitted to hospital outside of normal weekday working hours or at weekends (the ‘weekend effect’). Some studies have shown that acute stroke patients admitted at weekends have lower quality of care^1 2^ and higher mortality,^1–10^ while others have shown the opposite.^11–14^ Evaluation of these studies is further complicated by recent evidence that stroke incidence reporting at the weekend may be unreliable in older studies.^15^ Recent work based on data from the Sentinel Stroke National Audit Programme (SSNAP) data set further shows that care quality and outcomes in acute stroke vary across the week, and concluded that binary comparisons of weekend versus weekday or in-hours versus out-of-hours processes and effects oversimplify more likely variations by day of week and time of day.^16^ Further, no studies have investigated the impact of time of admission on disability following a stroke.

If there is lower quality of care and there are worse outcomes at the weekend these could be linked to reduced staffing levels^17^; for acute stroke care, nurse staffing levels at weekends has been shown to be a significant predictor of mortality,^18^ while evidence from the USA suggests that specialised stroke units, with round-the-clock availability of specialist stroke teams and rapid access to imaging and thrombolysis, reduce variation in quality of care and outcomes across the week.^19–21^

In 2010 London centralised its acute stroke services using a hub-and-spoke network model.^22–24^ Out of 34 hospitals that had historically provided acute stroke care,^25^ eight were selected as host sites for hyperacute stroke units (HASUs). The HASU model involved the London Ambulance Service taking all patients with suspected stroke symptom onset within 48 hours to one of the eight HASUs.^26^ HASUs receive patients with suspected stroke and routinely provide immediate assessment by specialised stroke assessment teams, access to immediate brain imaging and the immediate delivery of intravenous thrombolysis where appropriate. Acute stroke patients seen at other medical facilities were similarly transferred as an emergency to a HASU. The aim of the HASUs was to provide specialised care for all acute stroke patients during the first 72 hours after onset of stroke. After 72 hours, patients requiring ongoing inpatient treatment are transferred to one of the 24 acute stroke units in London linked to HASUs. Eight of these were in the same hospital trust as a HASU.^27^

Performance standards for HASUs, linked to payments, were initially set by Healthcare for London^28^ and subsequently the London Strategic Clinical Networks to maintain high quality of care across the HASU stay. Some standards were set to provide rapid access to time-critical ‘front door’ measures (eg, dysphagia screen within 4 hours of admission, brain scans within 1 hour, administration of thrombolysis to eligible patients^26^ within 60 min). Other standards were set with less stringent time constraints (eg, stroke specialist consultant physician assessment within 24 hours, physiotherapist assessment within 72 hours).

On average across all patients, the quality of acute stroke care in London increased as a result of the centralisation and was significantly higher than elsewhere in England on all measures analysed,^29^ and mortality decreased.^30^ Following these findings, the aim of this study was to investigate variations in the quality of acute stroke care and outcomes by day and time of admission in London HASUs and the rest of England. We used national audit data for all patients in England who had a stroke during a 12 month period recorded by the SSNAP.^31^ We hypothesised that there would be less variation across the week in care quality measures within London HASUs compared with the variation in the rest of England, and that this would also translate into less variation in outcomes in London HASUs.

## Methods

### Data and measures

We obtained anonymised patient-level data from the SSNAP,^31^ for all patients in England with a primary diagnosis of stroke (ischaemic stroke or primary intracerebral haemorrhage) between 1 January 2014 and 31 December 2014. SSNAP collects data on clinical characteristics, care quality (from the time of admission up to 6 months after stroke) and outcomes for all stroke patients admitted to acute care hospitals in England.^32–34^ During our study period the case ascertainment in the SSNAP, which is calculated as the proportion of all acute stroke patients admitted to hospitals, for England was estimated to be 90%.^35^ We excluded patients treated at hospitals in Wales from our analysis because for Wales the case ascertainment was estimated to be 60%.^33^

The following quality of care indicators were measured from time of hospital admission (or onset of stroke symptoms for those who were already in hospital): brain scan within 1 hour and within 12 hours, dysphagia screen within 4 hours, assessment by a nurse trained in stroke management within 24 hours, administration of intravenous thrombolysis to eligible patients, door-to-needle time within 1 hour in patients receiving thrombolysis, assessment by a stroke specialist consultant physician within 12 hours* and within 24 hours, admission to a stroke unit within 4 hours, assessments by a physiotherapist within 24 hours* and within 72 hours, assessments by occupational therapist within 24 hours* and within 72 hours and assessments by speech and language therapist within 24 hours* and within 72 hours. These measures are quality indicators routinely reported by SSNAP; we also included measures (marked with a *) with more stringent time constraints to reflect the time-critical nature of acute stroke care. Outcomes were measured as whether or not the patient died within 3 days and disability using the modified Rankin Scale (mRS) score 0 to 2 versus 3 to 6 (moderate, moderately severe or severe disability or death) at the end of the inpatient stay. We also analysed mRS score 0 to 2 versus 3 to 5 at the end of the inpatient stay, excluding patients who died. Mortality data beyond hospital discharge were not available in SSNAP; we therefore measured mortality up to 3 days after admission to minimise the number of missed deaths. We analysed length of stay (LOS) in the HASU (in London only) and LOS in hospital. The denominators used for each measure were consistent with the SSNAP key indicators.^36^ Most outcomes were measured for all patients, but there were exceptions: patients who were medically unwell or refused to be screened were excluded from the dysphagia screen measure, only patients with ischaemic stroke who met the Royal College of Physicians guideline minimum threshold for thrombolysis were included in the thrombolysis rate, door-to-needle times included only those who received thrombolysis with a final diagnosis of stroke and patients who were persistently medically unwell, declined to be assessed or had no relevant deficit were excluded from the therapy performance measures.

To examine variations across the week we initially used a flexible specification of time of admission, measured in six 4 hour periods from 00:00 to 03:59, 04:00 to 07:59, 08:00 to 11:59, 12:00 to 15:59, 16:00 to 19:59, 20:00 to 23:59 for every day of the week (42 periods). We also created a more restrictive measure to examine broad trends across the week: Monday to Friday 08:00 to 19:59, Monday to Friday 20:00 to 07.59, Saturday and Sunday 08:00 to 19:59, Saturday and Sunday 20:00 to 07.59 (four periods) following Bray *et al* 16 who found variations across the week with both specifications.

### Statistical analysis

We ran patient-level logistic regressions, regressing each measure against time period of admission. For LOS we used parametric survival models (modelled as time to event of discharge) assuming a lognormal survival distribution. We ran separate models for London and the rest of England. In every model we controlled for sex, age (continuous variable), ethnic group (six categories), type of stroke (infarction or primary intracerebral haemorrhage), comorbidities prior to admission (five options), mRS before stroke (0 to 2, 3 to 5), level of consciousness on arrival at the hospital (four categories), method of admission to the hospital (three categories), time from onset of stroke symptoms to admission (four categories), month of admission (12 categories) and hospital trust. When analysing mRS scores 0 to 2 versus 3 to 5 at the end of the inpatient spell we additionally controlled for the number of days after admission at which the mRS score was measured. We were unable to do this for the analysis of mRS score 0 to 2 versus 3 to 6 as date of death was not available. We tested for statistically significant variations across the week using Wald tests and reported the results as joint p values under the null hypothesis that the regression coefficients for every time period relative to the omitted time period were zero. We calculated the average predicted probability of each outcome (predicted median LOS in the case of the LOS variables) in each time period controlling for the covariates. Patients admitted with a diagnosis of acute stroke in London who were not treated in a HASU were excluded (6% all London patients in our data set were not treated in a HASU). P values<0.05 were considered to be statistically significant. Data on National Institutes of Health Stroke Scale (NIHSS) score, a validated measure of stroke severity on a scale from 0 (no stroke symptoms) to 42 (severe stroke), were available for 93% patients in London HASUs and 77% patients in the rest of England. Due to the extent of missing NIHSS data, in our main analysis we controlled for stroke severity using level of consciousness on arrival at the hospital (one component of NIHSS); we then reran all analyses controlling for NIHSS on arrival at the hospital on the smaller sample instead of level of consciousness on arrival. The findings using NIHSS score on arrival were qualitatively the same and are presented in the online supplementary figures S1-S6.

10.1136/bmjopen-2018-025366.supp1 Supplementary data

### Patient and public involvement

Two stroke patient representatives contributed to the design of our study protocol and development of the research questions; they also contributed to discussions of interim findings presented at study steering committee meetings in June 2015 and July 2016, raising issues related to variation in quality of care and mortality, which we incorporated into our analysis. They were consulted on the methods for disseminating the outputs of this study and ensured that we were addressing questions and communicating lessons in a meaningful way. The findings of this research will be disseminated to the relevant patient community in an accessible way.

## Results

**Figure 1:**
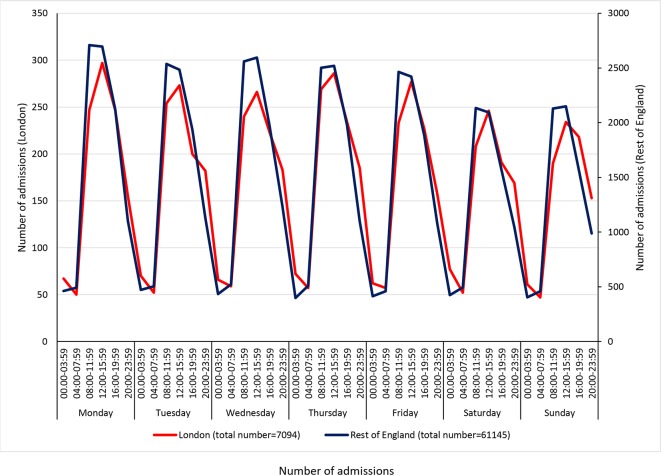
Number of admissions in London and rest of England across the 42 time periods in the week. Note: Left-hand y-axis relates to London HASUs, right-hand y-axis to the rest of England. Shaded areas indicate 20:00 to 07:59 each day of the week. HASU, hyperacute stroke units.

The study cohort comprised 68 239 patients (7094 from London HASUs, 61 145 from the rest of England) from 208 hospitals (eight London HASUs, 200 hospitals from the rest of England). The number of admissions varied across the week, with similar trends for London HASUs and the rest of England: there were more admissions during the day than at night, more admissions in the day during the week compared with during the day at the weekend, similar numbers of admissions during the night each day and the highest number of admissions was during the day on Monday (figure 1). In London HASUs the total number of admissions across all hospitals during the 12 month period ranged from 47 to 297 across the 42 time periods; in the rest of England it ranged from 398 to 2709. There was slightly higher proportion of men than women in London compared with the rest of England, the mean age was slightly lower and patients were less likely to be white (all p values<0.001; table 1). There were also differences in the pattern of pre-existing comorbidities, London HASUs case mix was characterised by a larger proportion of people having congestive heart failure, hypertension and diabetes, while in the rest of England, patients were more likely to have atrial fibrillation and previously have had a stroke or TIA (all p values<0.001). The mRS before stroke was higher in London HASUs compared with the rest of England, suggesting there were more people with at least moderate disability (<0.001). A higher proportion of patients arrived to the hospital in an ambulance in London compared with the rest of England (<0.001). A slightly higher proportion of patients was admitted to the hospital in London compared with the rest of England within more than 6 hours from onset of stroke symptoms, but the proportion of the patients with unknown time of symptoms’ onset was also lower in London (<0.001).

**Table 1:** Patient characteristics

	London HASUs (n=7094)	Rest of England (n=61 145)	Difference	P value*
Sex				0.0001
Male	3719 (52%)	30 536 (50%)	2%	
Female	3375 (48%)	30 609 (50%)	−2%	
Age, years (mean (SD))	72 (15)	75 (13)	−3 years	<0.0001
Ethnic group				<0.0001
White	4332 (61%)	56 221 (92%)	−31%	
Mixed	72 (1%)	141 (<1%)	<1%	
Black	650 (9%)	1272 (2%)	7%	
Asian	505 (7%)	362 (<1%)	6%	
Other	526 (7%)	358 (<1%)	6%	
Not available	1009 (14%)	2791 (5%)	9%	
Type of stroke				0.0531
Infarction	6252 (88%)	54 355 (89%)	−1%	
Primary intracerebral haemorrhage	842 (12%)	6790 (11%)	1%	
Comorbidities prior to admission				
Congestive heart failure	439 (6%)	3204 (5%)	1%	0.0008
Hypertension	4284 (60%)	32 447 (53%)	7%	<0.0001
Atrial fibrillation	1229 (17%)	12 655 (21%)	−4%	<0.0001
Diabetes	1705 (24%)	12 024 (20%)	4%	<0.0001
Stroke/TIA	1688 (24%)	16 752 (27%)	−4%	<0.0001
mRS score before stroke				<0.0001
Slight or no disability (0–2)	5552 (78%)	49 574 (81%)	−3%	
At least moderate disability (3-5)	1542 (22%)	11 571 (19%)	3%	
Level of consciousness on arrival at the hospital†				0.0263
Alert	5991 (84%)	51 230 (84%)	0%	
Not alert, but respond to minor stimulation	663 (9%)	5724 (9%)	0%	
Not alert, requires repeated stimulation	281 (4%)	2438 (4%)	0%	
Unresponsive	159 (2%)	1753 (3%)	−1%	
NIHSS on arrival at the hospital, score (median (IQR))	5 (2–11)	4 (2–9)	1 IQR	<0.0001
Method of admission to the hospital				<0.0001
Already inpatient	173 (2%)	3288 (5%)	−3%	
Ambulance	5966 (84%)	47 096 (77%)	7%	
Walk-in	955 (14%)	10 761 (18%)	−4%	
Time from onset of stroke symptoms to admission				<0.0001
<180 min	2741 (39%)	24 233 (40%)	−1%	
180–359 min	759 (11%)	5871 (10%)	1%	
≥360 min	1516 (21%)	10 773 (18%)	3%	
Time of onset not known	2078 (29%)	20 268 (33%)	4%	

Figures are n(%) except for age, which is mean (SD), and NIHSS on arrival at the hospital, which is median (IQR). The sample with NIHSS scores on arrival was n=6571 in London HASUs and n=47 126 in the rest of England.

†Level of consciousness scores taken from admission NIHSS score (Question 1a).

*P value threshold adjusted for multiple testing is 0.0038.

HASU, hyperacute stroke units; NIHSS, National Institutes of Health Stroke Scale; mRS, modified Rankin Scale; TIA, transient ischaemic attack.

**Figure 2:**
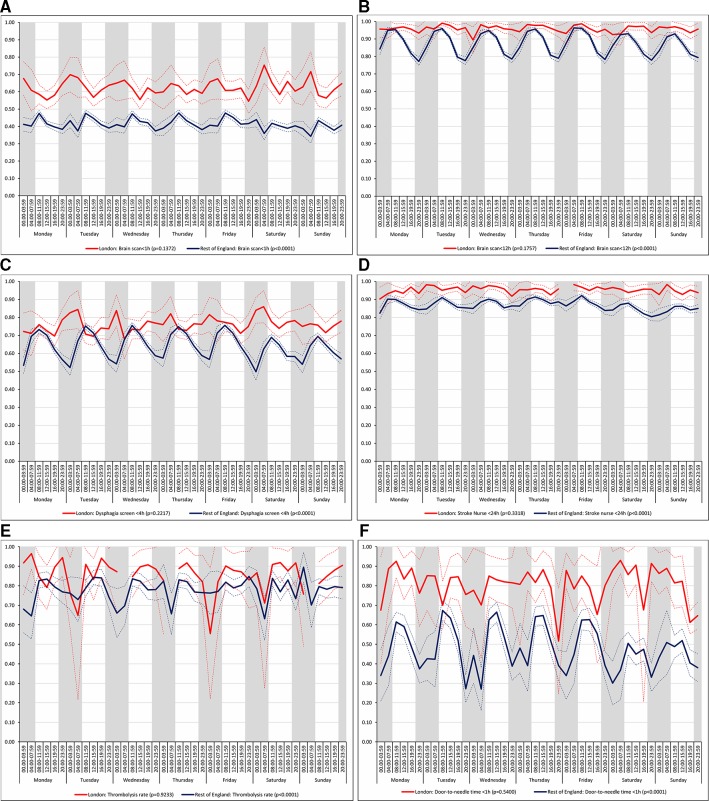
Quality of care across the 42 time periods in the week: measures linked to performance standards for London HASUs (A) brain scan within 1 hour, (B) brain scan within 12 hours, (C) dysphagia screen within 4 hours, (D) assessment by a nurse trained in stroke management within 24 hours, (E) administration of intravenous thrombolysis to eligible patients and (F) door-to-needle time within 1 hour in patients receiving thrombolysis. Note: Figures are average predicted probabilities of each outcome in each time period controlling for the covariates. Dashed lines represent lower and upper 95% confidence limits. Shaded areas indicate 20:00 to 07:59 each day of the week. P values indicate significant variation (p<0.05) or not for each measure over the week in each region. Gaps in the solid line indicate that all patients in that time period achieved that outcome. HASU, hyperacute stroke units.

There was no significant variation in care quality across the 42 time periods in any of the measures relating to brain scanning, stroke nursing care and thrombolysis in London HASUs (all p values>0.05), but there was significant variation in these measures in the rest of England (all p values<0.001; figure 2). For each measure in the rest of England there was variation by time of day every day, with the likelihood of receiving these interventions worse for patients admitted at night.

**Figure 3:**
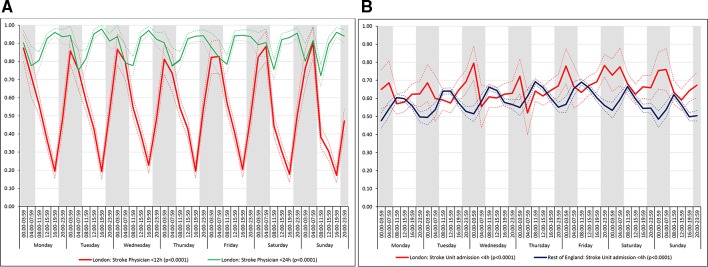
Quality of care across the 42 time periods in the week: variation by time of day but not day of the week (A) assessment by a stroke specialist consultant physician within 12 and 24 hours in London HASUs and (B) admission to a stroke unit within 4 hours. Note: Figures are average predicted probabilities of each outcome in each time period controlling for the covariates. Dashed lines represent lower and upper 95% confidence limits. Shaded areas indicate 20:00 to 07:59 each day of the week. P values indicate significant variation (p<0.05) or not for each measure over the week in each region. Figure 3A includes two measures for London HASUs. HASU, hyperacute stroke units.

**Figure 4:**
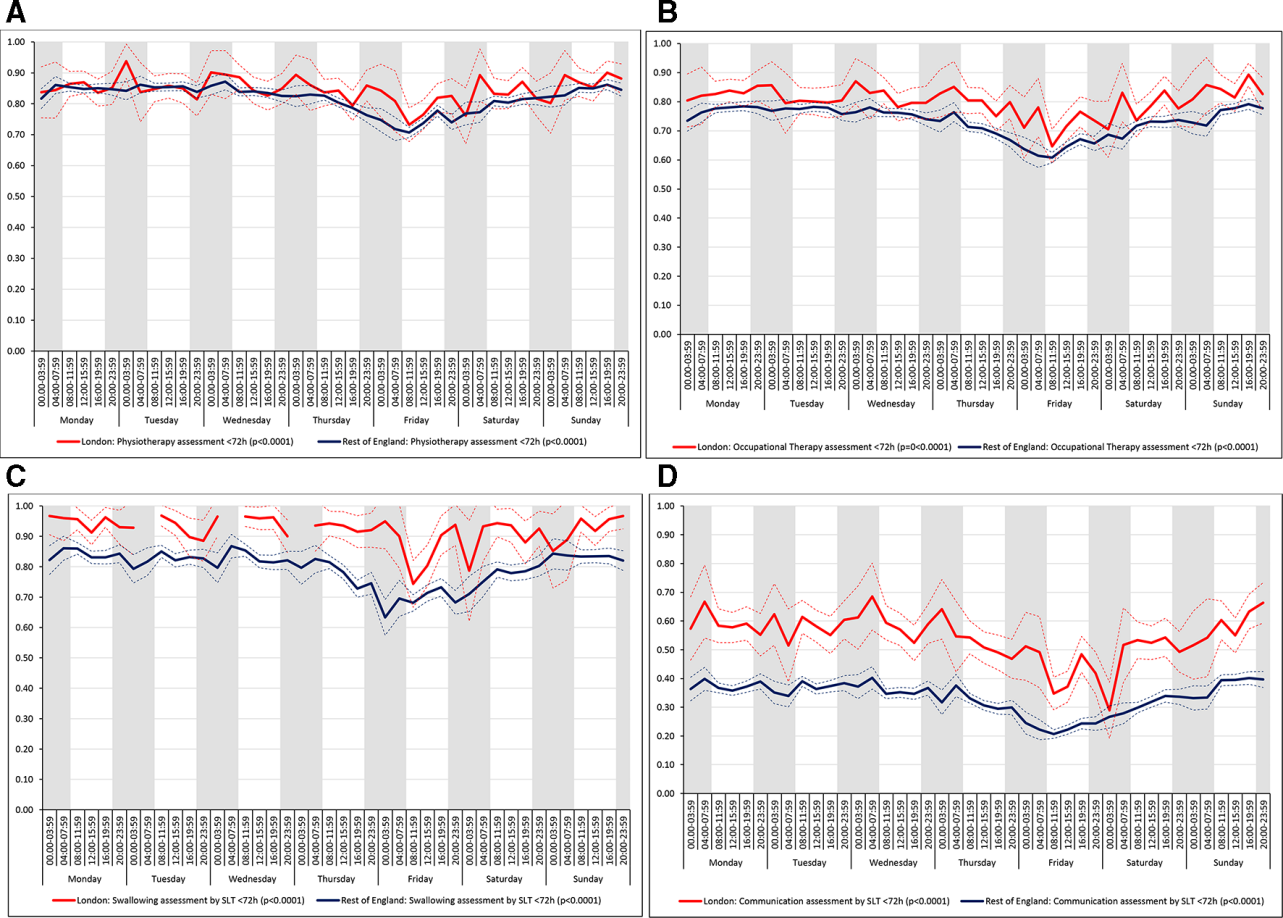
Quality of care across the 42 time periods in the week: variation by day of the week but not time of day (A) physiotherapist assessment within 72 hours, (B) occupational therapist assessment within 72 hours, (C) swallow assessment by a speech and language therapist within 72 hours and (D) communication assessment by a speech and language therapist within 72 hours. Note: Figures are average predicted probabilities of each outcome in each time period controlling for the covariates. Dashed lines represent lower and upper 95% confidence limits. Shaded areas indicate 20:00 to 07:59 each day of the week. P values indicate significant variation (p<0.05) or not for each measure over the week in each region. Gaps in the solid line indicate that no patients in that time period achieved that outcome.SLT, speech and language therapist.

**Figure 5:**
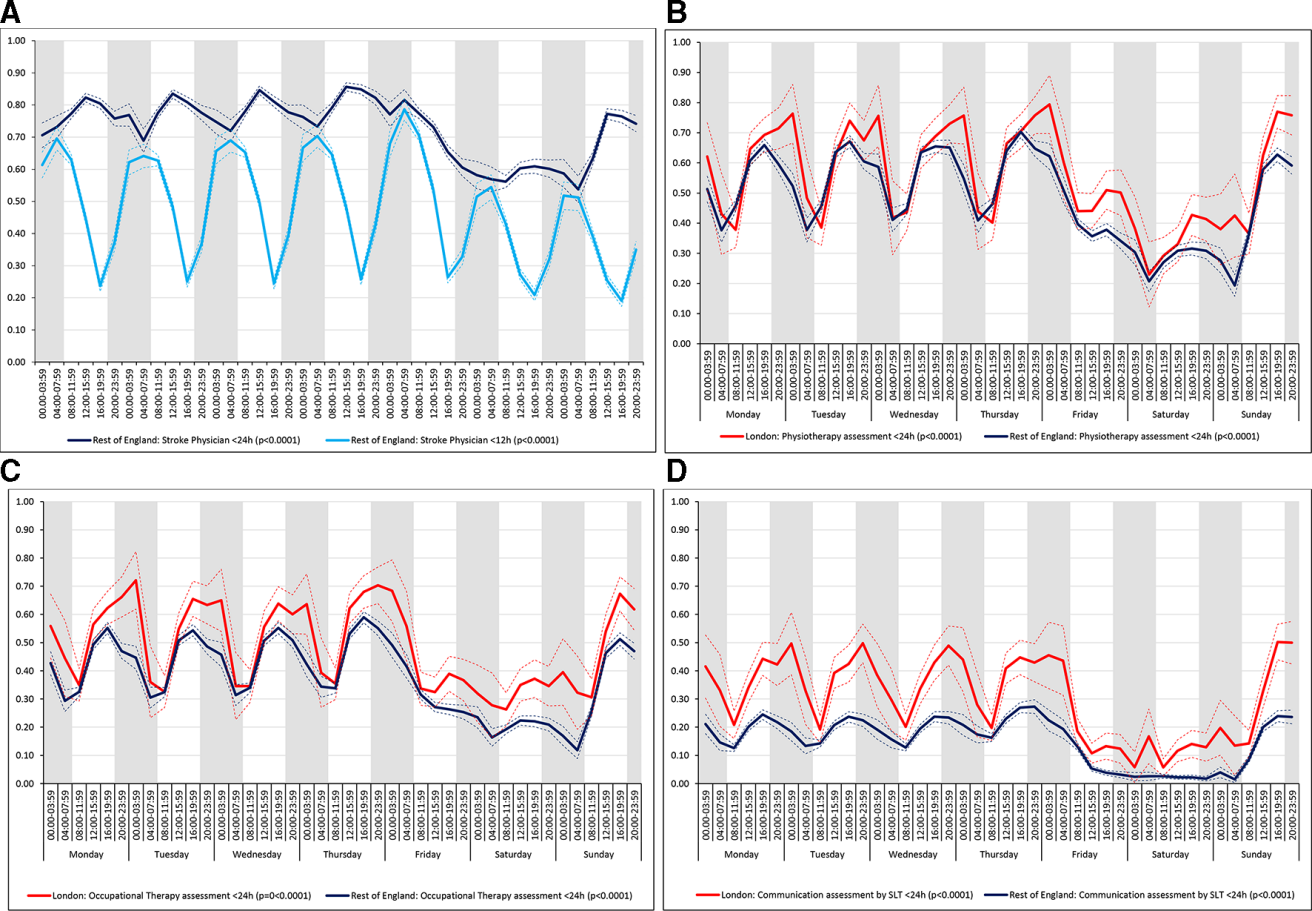
Quality of care across the 42 time periods in the week: variation by time of day and day of the week (A) assessment by a stroke specialist consultant physician within 12 and 24 hours in rest of England, (B) physiotherapist assessment within 24 hours, (C) occupational therapist assessment within 24 hours and (D) communication assessment by a speech and language therapist within 24 hours. Note: Figures are average predicted probabilities of each outcome in each time period controlling for the covariates. Dashed lines represent lower and upper 95% confidence limits. Shaded areas indicate 20:00 to 07:59 each day of the week. P values indicate significant variation (p<0.05) or not for each measure over the week in each region. Figure 5A includes two measures for rest of England.SLT, speech and language therapist.

For all the other quality of care measures there was significant variation by time period of admission across the week both in London and the rest of England (all p values<0.001). There were three patterns of variation: (1) Variation by time of day but not day of the week was observed for assessment by a stroke specialist consultant physician within 12 hours and within 24 hours in London HASUs and admission to a stroke unit within 4 hours in London and the rest of England (figure 3). With this pattern similar variations during the day were found each day of the week. (2) Variation by day of the week but not time of day was observed for assessments by a physiotherapist, occupational therapist and speech and language therapist within 72 hours in London HASUs and the rest of England (figure 4). With this pattern care quality was worse for patients admitted on Friday. (3) Variation by time of day and day of the week was observed for assessment by a stroke specialist consultant physician within 12 hours and within 24 hours in the rest of England and for therapist assessments within 24 hours in London HASUs and the rest of England (figure 5). With this pattern, there was variation during the day on Monday to Friday and care quality was worse at weekends.

**Figure 6:**
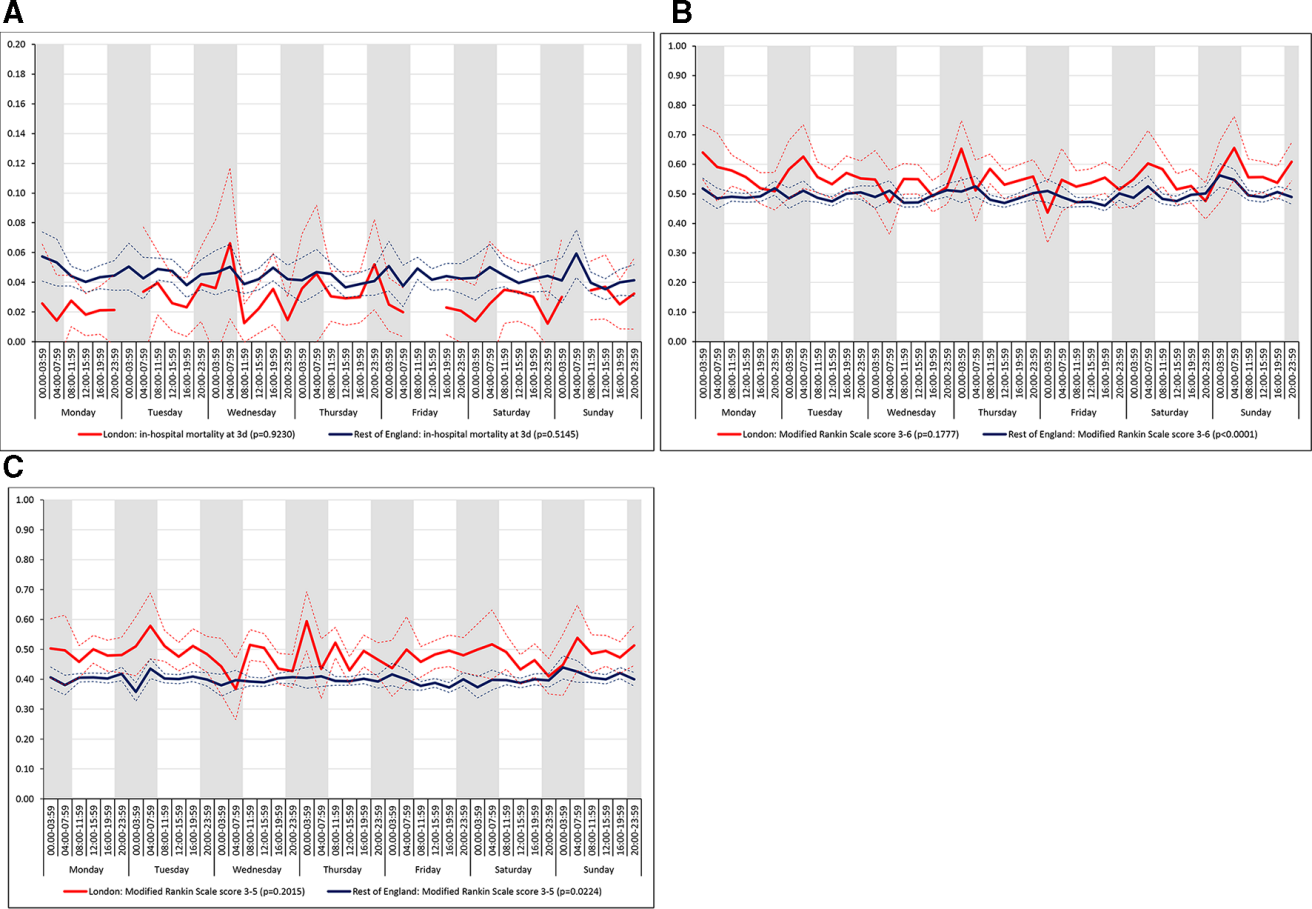
Outcomes across the 42 time periods in the week (A) mortality at 3 days, (B) modified Rankin Scale score 3 to 6 and (C) modified Rankin Scale score 3 to 5*. Note: Figures are average predicted probabilities of each outcome in each time period controlling for the covariates. Dashed lines represent lower and upper 95% confidence limits. Shaded areas indicate 20:00 to 07:59 each day of the week. P values indicate significant variation (p<0.05) or not for each measure over the week in each region. Gaps in the solid line indicate that no patients in that time period achieved that outcome. Note, the scaling of the y-axis in figure 6A is not from zero to one.

There was no significant variation in outcomes across the 42 time periods in London HASUs (all p values>0.05; figure 6A). In the rest of England there was significant variation in disability (p value<0.001 for mRS scores 0 to 6, and p value=0.022 for mRS scores 0 to 5), figure 6B and C) but not mortality (p value>0.05); mRS scores at the end of the inpatient episode varied by time of admission on every day and were worse among patients admitted at night. It is worth noting that, based on the point estimates in each period, it appears there is more variation in mRS scores in London HASUs. One reason why the variation in London HASUs was not statistically significant might be because of the larger uncertainty at each time point.

**Figure 7:**
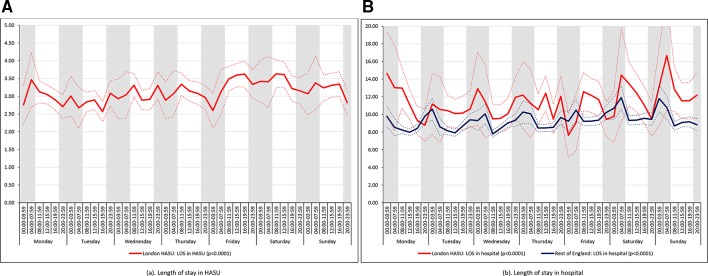
Length of stay across the 42 time periods in the week (A) length of stay in HASU and (B) length of stay in hospital. Note: Figures are average predicted probabilities of each outcome in each time period controlling for the covariates. Dashed lines represent lower and upper 95% confidence limits. Shaded areas indicate 20:00 to 07:59 each day of the week. P values indicate significant variation (p<0.05) or not for each measure over the week in each region.

There was significant variation in LOS across the 42 time periods in London HASUs and the rest of England both in terms of HASU LOS and total inpatient LOS (p value<0.001 for London HASUs LOS, p value=0.005 for total LOS in London hospitals and p values<0.001 for LOS in the rest of England hospitals; figure 7). Median HASU LOS in London varied between 2.6 and 3.6 days across the 42 time periods. It was difficult to detect a trend by day and time of admission in London HASU LOS and inpatient LOS. In the rest of England median inpatient LOS was longer among those admitted at night.

Results using the four time period specification (table 2) were broadly similar to those with the 42 time periods, but pooling time periods meant that the extent of variation during the week for some of the quality of care measures was reduced (for unadjusted figures and p values, see online supplementary table 1 and online supplementary table 2, respectively). In these analyses there was no significant variation in London in quality of care measures linked to specialist stroke nurse assessments, rapid access to brain scans and administration of thrombolysis to eligible patients for London HASUs, nor was there in the outcome measures. With the exception of mortality at 3 days and mRS scores 3 to 5 at the end of the inpatient spell, all of these measured varied significantly in the rest of England. LOS varied significantly for London HASUs and the rest of England; for London HASUs pooling time periods more clearly indicates longer LOS among patents admitted at the weekend; for the rest of England the trends were as in the 42 time period model, with longer LOS among patients admitted at night.

**Table 2:** Quality of care and outcomes across four periods in the week

	London HASUs					Rest of England				
	Weekday	Weekend	Weekday	Weekend	P values*	Weekday	Weekend	Weekday	Weekend	P values*
	08:00-19:59	08:00-19:59	20:00-07:59	20:00-07:59		08:00-19:59	08:00-19:59	20:00-07:59	20:00-07:59	
Quality of care measures										
Brain scan within 1 hour	0.60 (0.58–0.61)	0.61 (0.58–0.63)	0.63 (0.60–0.65)	0.65 (0.61–0.68)	0.0344	0.44 (0.44–0.45)	0.41 (0.40–0.41)	0.40 (0.39–0.40)	0.39 (0.38–0.41)	<0.0001
Brain scan within 12 hours	0.97 (0.96–0.97)	0.96 (0.95–0.97)	0.95 (0.94–0.96)	0.95 (0.93–0.96)	0.0093	0.90 (0.90–0.90)	0.88 (0.87–0.89)	0.84 (0.83–0.84)	0.83 (0.82–0.84)	<0.0001
Dysphagia screen within 4 hours	0.74 (0.72–0.75)	0.75 (0.73–0.77)	0.77 (0.75–0.79)	0.79 (0.76–0.82)	0.0029	0.70 (0.70–0.71)	0.65 (0.64–0.66)	0.60 (0.59–0.61)	0.58 (0.56–0.59)	<0.0001
Assessment by a nurse trained in stroke management within 24 hours	0.96 (0.95–0.96)	0.94 (0.93–0.96)	0.95 (0.94–0.96)	0.95 (0.94–0.97)	0.1872	0.89 (0.88–0.89)	0.85 (0.85–0.86)	0.86 (0.86–0.87)	0.83 (0.82–0.84)	<0.0001
Administration of intravenous thrombolysis to eligible patients	0.88 (0.86–0.90)	0.88 (0.84–0.92)	0.86 (0.82–0.91)	0.88 (0.82–0.95)	0.9327	0.81 (0.80–0.82)	0.80 (0.78–0.82)	0.76 (0.74–0.78)	0.76 (0.72–0.79)	<0.0001
Door-to-needle time within 1 hour in patients receiving thrombolysis	0.84 (0.81–0.87)	0.89 (0.85–0.93)	0.79 (0.74–0.84)	0.84 (0.77–0.91)	0.0269	0.60 (0.59–0.62)	0.48 (0.45–0.50)	0.38 (0.35–0.40)	0.37 (0.33–0.41)	<0.0001
Assessment by a stroke specialist consultant physician within 12 hours	0.39 (0.38-.40)	0.30 (0.27–0.32)	0.63 (0.61–0.66)	0.64 (0.60–0.68)	<0.0001	0.48 (0.48–0.49)	0.30 (0.29–0.31)	0.51 (0.51–0.52)	0.42 (0.41–0.44)	<0.0001
Assessment by a stroke specialist consultant physician within 24 hours	0.90 (0.89–0.91)	0.87 (0.85–0.89)	0.90 (0.88–0.91)	0.92 (0.90–0.94)	0.0043	0.80 (0.79–0.80)	0.65 (0.65–0.66)	0.75 (0.74–0.75)	0.62 (0.61–0.64)	<0.0001
Admission to a stroke unit within 4 hours	0.62 (0.60–0.63)	0.64 (0.61–0.66)	0.67 (0.65–0.70)	0.70 (0.67–0.74)	<0.0001	0.63 (0.63–0.63)	0.59 (0.58–0.60)	0.55 (0.54–0.56)	0.53 (0.52–0.55)	<0.0001
Physiotherapist assessment within 72 hours	0.83 (0.82–0.84)	0.86 (0.84–0.88)	0.85 (0.83–0.87)	0.84 (0.81–0.87)	0.0693	0.82 (0.81–0.82)	0.83 (0.82–0.84)	0.81 (0.81–0.82)	0.82 (0.80–0.83)	0.0010
Occupational therapist assessment within 72 hours	0.79 (0.78–0.80)	0.82 (0.80–0.84)	0.81 (0.79–0.82)	0.80 (0.76–0.83)	0.0967	0.73 (0.73–0.74)	0.75 (0.75–0.76)	0.73 (0.72–0.74)	0.73 (0.72–0.74)	<0.0001
Swallow assessment by a SLT within 72 hours	0.92 (0.91–0.93)	0.93 (0.91–0.95)	0.93 (0.91–0.95)	0.91 (0.88–0.95)	0.5838	0.80 (0.80–0.81)	0.81 (0.80–0.82)	0.79 (0.78–0.80)	0.80 (0.78–0.82)	0.0946
Communication assessment by a SLT within 72 hours	0.53 (0.51–0.54)	0.56 (0.54–0.59)	0.55 (0.53–0.58)	0.52 (0.48–0.56)	0.0739	0.33 (0.32–0.33)	0.36 (0.35–0.37)	0.34 (0.33–0.35)	0.34 (0.32–0.35)	<0.0001
Physiotherapist assessment within 24 hours	0.56 (0.54–0.57)	0.47 (0.45–0.50)	0.65 (0.63–0.68)	0.48 (0.44–0.52)	<0.0001	0.54 (0.54–0.55)	0.41 (0.40–0.41)	0.53 (0.52–0.54)	0.35 (0.34–0.37)	<0.0001
Occupational therapist assessment within 24 hours	0.49 (0.47–0.50)	0.42 (0.40–0.45)	0.58 (0.55–0.60)	0.41 (0.37–0.45)	<0.0001	0.43 (0.42–0.43)	0.31 (0.30–0.31)	0.42 (0.42–0.43)	0.26 (0.25–0.27)	<0.0001
Communication assessment by a SLT within 24 hours	0.29 (0.28–0.31)	0.22 (0.20–0.24)	0.39 (0.37–0.42)	0.23 (0.20–0.27)	<0.0001	0.17 (0.17–0.17)	0.10 (0.09–0.10)	0.19 (0.18–0.20)	0.08 (0.07–0.09)	<0.0001
Outcome measures										
Mortality at 3 days	0.03 (0.02–0.03)	0.03 (0.02–0.04)	0.03 (0.02–0.04)	0.02 (0.01–0.03)	0.3310	0.04 (0.04–0.05)	0.04 (0.04–0.04)	0.05 (0.04–0.05)	0.05 (0.04–0.05)	0.1055
mRS score 3–6	0.55 (0.53–0.56)	0.55 (0.52–0.57)	0.55 (0.52–0.57)	0.56 (0.53–0.59)	0.8672	0.48 (0.48–0.48)	0.49 (0.48–0.50)	0.51 (0.50–0.51)	0.51 (0.50–0.52)	<0.0001
mRS score 3–5†	0.49 (0.47–0.50)	0.47 (0.45–0.50)	0.48 (0.45–0.50)	0.48 (0.44–0.51)	0.7497	0.40 (0.39–0.40)	0.40 (0.39–0.41)	0.40 (0.39–0.41)	0.40 (0.39–0.41)	0.3746
Length of stay										
Length of stay in HASU (days)	3.1 (3.0–3.2)	3.4 (3.2–3.5)	3.0 (2.9–3.1)	3.1 (2.9–3.3.)	0.0007					
Length of stay in hospital (days)	10.8 (10.2–11.3)	12.1 (11.1–13.1)	10.8 (10.0–11.7)	11.5 (10.2–12.9)	0.0359	8.5 (8.4–8.6)	9.2 (9.0–9.4)	9.7 (9.4–9.9)	10.1 (9.6–10.5)	<0.0001

Figures are average predicted probabilities (95% CIs) of each measure in each time period controlling for the covariates.

*P value threshold adjusted for multiple testing is 0.0025 for London HASUs and 0.0024 for the rest of England.

†Patients who died were not included.

HASU, hyperacute stroke units; mRS, modified Rankin Scale; SLT, speech and language therapist.

Results were similar when controlling for NIHSS score on arrival at hospital instead of level of consciousness on the smaller sample of patients with non-missing NIHSS data: results with p values<0.05 and trends across the week were unchanged (online supplementary figures S1-S6 and online table 3 in the supplementary materials).

## Discussion

### Principal findings

In our study, we found no evidence for an admission effect across the week on early outcomes in acute stroke patients admitted to a London HASU: 3-day mortality and modified Rankin Scale score at hospital discharge did not vary by day and time of admission in London HASUs. This is consistent with a recent study based on administrative data in the UK^9^ that found a steady reduction in in-hospital mortality difference between weekday and weekend stroke admissions in 2008 to 2014 across England and that this difference is no longer statistically significant in 2014.

There was also no variation by day and time of admission across the week in terms of rapid access to brain scanning, stroke nursing care and thrombolysis in London HASUs. Other quality of care measures did significantly vary across the week in London HASUs, and three patterns of variation were detected: by time of day but not day of the week, by day of the week but not time of day and by time of day and day of the week. LOS was longer among patients admitted to London HASUs at the weekend. In the rest of England there was variation in all measures by day and time of admission across the week, except for mortality at 3 days. We hypothesised there would be less variation across the week in care quality measures in London HASUs compared with the rest of England, and that this would translate into less variation in outcomes in London HASUs. The lower variation in care quality measures across the week in London HASUs was confirmed, but only with respect to ‘front door’ measures of acute stroke care. With respect to the health outcomes: there was no variation in mortality at 3 days and disability at hospital discharge by day and time of admission across the week in London HASUs. This is consistent with previous studies showing that timely access to thrombolysis is associated with good stroke outcomes.^37^ In the rest of England there was no variation in 3-day mortality by day and time of admission across the week (but there was in terms of disability after discharge), suggesting the lack of variation in outcomes in London HASUs may not be exclusively attributed to the lack of variation in ‘front door’ quality of care.

### Strengths and limitations

The main strength of our study is the large national data set we have used containing detailed information on quality of care, outcomes and patient characteristics. We have examined whether time of admission was related to quality of care using a comprehensive set of indicators from across the acute stroke care pathway. Most of the measures were from a pre-existing set of national acute stroke care indicators, and those that were added had more stringent time constraints to reflect the time-critical nature of acute stroke care. Our outcomes were stroke mortality and disability, where previous studies have focused on mortality.^2 4 5 7–10^ The rich set of patient characteristics in the data set meant we could control for patient factors likely to affect quality of care and outcomes that vary by day and time of admission across the week and between London and the rest of England. There are several limitations. First, while case ascertainment in SSNAP was 90% during the time period of our study, these data might not be representative of all stroke patients. For example, not all hospitals receiving acute stroke patients in England participated in SSNAP, and the results may not be representative of hospitals who did not participate. Second, while analyses of hospital administrative data to investigate weekend effects in stroke have been undermined by evidence of variations in inaccurate coding across the week,^15^ in SSNAP data are inputted voluntarily by hospitals and we cannot exclude the possibility of inaccurate or selective reporting. Particularly problematic for our study would be if this bias was more likely to occur in London or the rest of England and/or if it was more likely to vary by time of admission. Third, we were unable to measure long-term outcomes as these were not available in SSNAP. Mortality data in SSNAP are currently only available for patients who are in hospital and therefore to reduce the risk of bias we measured mortality at 3 days after admission when most patients will still be admitted. Three-day mortality has been used in previous studies to evaluate the centralisation of acute stroke services in London,^30^ but the focus in our study on in-hospital mortality only is a further limitation. Similarly, long-term disability data are not reliably collected in SSNAP, and so this was measured by mRS at the end of the inpatient spell. Fourth, while the richness of our data set means we have been able to control for confounding factors we cannot exclude the possibility of confounding due to unobserved patient characteristics or staffing levels. Fifth, while the sample size of our study is large in both London and the rest of England, when evaluating quality of care and outcomes across the week the number of observations in each time period was considerably smaller in London. We cannot exclude the possibility that the smaller number of patients in London resulted in wider CIs around the adjusted predicted probabilities in each time period making it less likely to show significant variation in the measures evaluated.

### Comparison with other studies

There is a large literature examining weekend effects in healthcare across a range of clinical areas.^38^ In acute stroke there is conflicting evidence as to whether patients admitted at weekends have higher or lower quality of care and better or worse outcomes,^1–8^ but recent analyses have shown that care quality and outcomes in acute stroke vary across the week, and that comparing weekend versus weekday or in-hours versus out-of-hours effects is flawed as it does not take into account variations by day of the week and time of day.^16^ This study, using the same data set as ours but from an earlier time period and analysing the whole of England and Wales, found that quality of care varied across the entire week, not only between weekends and weekdays, with a number quality of care measures showing different patterns of variation over the week. While the findings mirrored our own for the rest of England, one noticeable difference was in mortality: Bray *et al* reported that patients admitted overnight on weekdays had lower odds of survival (0.90, 95% CI 0.82 to 0.99) compared with those admitted during the day at weekdays; this difference might be because our survival measure is not the same (3 vs 30 days) and/or because our extract of the SSNAP data set is more recent. What our study adds is analyses of variation in quality of care and outcomes in London HASUs separately following the centralisation of acute stroke services in London in 2010, which has been shown to increase the quality of care and outcomes on average across the week.^29 30^ Our findings were further expanded in Black *et al* 39 that aimed to identify factors influencing this variation.^39^

### Implications

There are several implications of our study. The first is that London HASUs appear to operate a uniform service across the week with respect to some but not all aspects of acute stroke care. Performance standards originally set by Healthcare for London stipulated that London HASUs should operate a 24/7 service with respect to first assessment by a stroke nurse, rapid access to brain scans and administration of thrombolysis to eligible patients; our findings show that London HASUs do operate a 24/7 service with respect to these measures. However, for other less time-critical measures, such as senior stroke physician assessment within 24 hours and therapist assessments within 72 hours, we found significant variation by day and time of admission across the week in London HASUs. This suggests that some performance standards like ‘front door’ interventions may be emphasised more than others and analysis of qualitative data collected in Black *et al* 39 complemented our findings.^39^ The second implication is that there are differences in acute stroke care between London HASUs and the rest of England across the week, with less variation in quality of care and outcomes in London HASUs. The main differences were observed in nursing care, brain scanning and thrombolysis provision, and also with the type of variation observed for stroke consultant care. For these measures, our results show that the centralised model in London is more effective at providing constant care across the week. In terms of comparing London and the rest of England, four further issues are worth bearing in mind. First, our study focuses on patients admitted to London HASUs only, not other hospitals in London; our data suggest that 6% of acute stroke patients in London are not treated in a HASU. However, some of these patients will not have been eligible for HASU care because of greatly delayed presentation or identification of stroke, and others will have had a stroke after surgical procedures or in another context which precluded their admission to a HASU. Our focus on London HASUs was deliberate as the aim of our study was to evaluate the HASU model, but it means that our findings for London HASUs should not be generalised to all patients in London. Indeed, there is evidence that quality of care is lower for acute stroke patients in London not treated in a HASU compared with those who are.^29^ Second, and conversely, HASUs operate in many other parts of England using different models of care.^31 40^ In Greater Manchester, for instance, HASUs have also been shown to have higher quality of care than the rest of England excluding London.^29^ Hence the differences observed between London HASUs and the rest of England cannot be interpreted as a direct comparison of HASU versus non-HASU care, though if HASU-based care outside London was removed from the rest of England then the differences observed in this study are likely to be the same or greater. The third issue is that the London model may not apply to services operating in rural settings — in particular the greater travel times in rural areas make centralisation challenging.^41^ This means that potential benefits of the London model in terms of 24/7 care are unlikely to be achieved nationwide. The fourth issue is that the centralisation of acute stroke services in London was estimated to occur at an additional cost of £20 million, allocated to cover the increased cost per bed day in a HASU.^28^ With this additional level of funding it might be expected that the quality of care in London should improve, though whether it should produce less variation in quality of care and outcomes across the week in London compared with the rest of England depends on the relative levels of funding in both areas. There is some evidence that the reorganisation in London was cost-effective,^42 43^ but further analyses accounting for the size of the upfront investment, the relatively high costs per day of hyperacute stroke care, the impact on mortality and disability and the lifetime costs incurred by the NHS, social services and families caring for stroke survivors at different levels of disability would be helpful.

### Future research

Further research would be beneficial to evaluate the impact of stroke admission at different times of the week on longer-term mortality and disability outcomes, and to investigate the relationship between quality of care and outcomes and if this relationship varies by time of admission. Further research would also be useful to investigate the reasons for the differences in variation found between London HASUs and the rest of England, and why for some standards care in London HASUs was constant across the week, irrespective of day and time of admission, but for others it was not. Performing follow-up studies to monitor attainment of key quality indicators and outcomes, complementary to the SSNAP clinical audit annual reports,^33 44^ would also be beneficial in order to get an overall picture of national trends and dynamics over time and look in detail at underlying reasons for that to understand what amendments to clinical guideline for stoke care ought to be proposed in the future. Also, accounting for the organisational factors at the stroke unit level could explain an important part of the variation in quality of acute stroke care and outcomes by day and time of admission in London HASUs and the rest of England. This research would help to further inform how acute stroke services ought to be designed in future to maximise patient outcomes in a cost-effective manner.
