# Supplementary material for: Variation in quality of acute stroke care by day and time of admission: prospective cohort study of weekday and weekend centralised hyperacute stroke unit care and non-centralised services
Source: BMJ Open. 2019 Nov 7;9(11):e025366. doi: 10.1136/bmjopen-2018-025366 (PMC6858222; doi:10.1136/bmjopen-2018-025366)
Supplement: Supplementary data [file bmjopen-2018-025366supp001.pdf]

Supplementary Tables

Supplementary Table 1. Quality of care and outcomes across four periods in the week unadjusted figures

	**London HASUs**					**Rest of England**				
	**Weekday**	**Weekend**	**Weekday**	**Weekend**	**p-value†**	**Weekday**	**Weekend**	**Weekday**	**Weekend**	**p-value†**
	**08:00-19:59**	**08:00-19:59**	**20:00-07:59**	**20:00-07:59**		**08:00-19:59**	**08:00-19:59**	**20:00-07:59**	**20:00-07:59**	
Quality of care measures that do not vary across the week in London HASUs										
Brain scan within one hour	0.58 (0.57-0.60)	0.62 (0.59-0.64)	0.61 (0.58-0.63)	0.63 (0.59-0.67)	0.0443	0.42 (0.41-0.43)	0.41 (0.40-0.42)	0.42 (0.41-0.43)	0.43 (0.42-0.45)	0.2145
Brain scan within 12 hours	0.95 (0.95-0.96)	0.96 (0.94-0.97)	0.93 (0.91-0.94)	0.92 (0.90-0.94)	0.0000	0.88 (0.88-0.89)	0.88 (0.87-0.88)	0.84 (0.83-0.84)	0.84 (0.83-0.85)	0.0000
Dysphagia screen within four hours	0.74 (0.72-0.75)	0.76 (0.73-0.78)	0.77 (0.74-0.79)	0.78 (0.74-0.81)	0.0359	0.69 (0.69-0.70)	0.66 (0.65-0.66)	0.61 (0.60-0.62)	0.60 (0.58-0.62)	0.0000
Assessment by a nurse trained in stroke management within 24 hours	0.96 (0.95-0.96)	0.95 (0.94-0.96)	0.95 (0.93-0.96)	0.95 (0.94-0.97)	0.4109	0.88 (0.88-0.89)	0.86 (0.85-0.86)	0.86 (0.85-0.86)	0.84 (0.82-0.85)	0.0000
Administration of intravenous thrombolysis to eligible patients	0.88 (0.85-0.90)	0.87 (0.83-0.92)	0.87 (0.83-0.91)	0.88 (0.81-0.95)	0.9905	0.80 (0.79-0.81)	0.79 (0.77-0.81)	0.74 (0.72-0.77)	0.75 (0.72-0.79)	0.0000
Door-to-needle time within one hour in patients receiving thrombolysis	0.84 (0.81-0.87)	0.88 (0.84-0.93)	0.79 (0.74-0.85)	0.85 (0.78-0.92)	0.0677	0.60 (0.59-0.62)	0.48 (0.45-0.51)	0.38 (0.35-0.41)	0.36 (0.32-0.41)	0.0000
Quality of care: measures that vary significantly across the week										
Assessment by a stroke specialist consultant physician within 12 hours	0.39 (0.37-.40)	0.30 (0.28-0.33)	0.63 (0.60-0.65)	0.65 (0.61-0.69)	0.0000	0.47 (0.47-0.48)	0.30 (0.30-0.31)	0.52 (0.51-0.53)	0.44 (0.42-0.45)	0.0000
Assessment by a stroke specialist consultant physician within 24 hours	0.90 (0.89-0.91)	0.87 (0.86-0.89)	0.90 (0.88-0.91)	0.92 (0.90-0.94)	0.0173	0.79 (0.79-0.80)	0.66 (0.65-0.67)	0.74 (0.73-0.75)	0.63 (0.62-0.65)	0.0000
Admission to a stroke unit within four hours	0.62 (0.60-0.63)	0.65 (0.62-0.67)	0.68 (0.66-0.70)	0.71 (0.67-0.74)	0.0000	0.62 (0.62-0.63)	0.60 (0.59-0.61)	0.55 (0.54-0.56)	0.55 (0.53-0.57)	0.0000
Physiotherapist assessment within 72 hours	0.83 (0.82-0.84)	0.86 (0.84-0.88)	0.85 (0.83-0.87)	0.84 (0.81-0.87)	0.0538	0.82 (0.82-0.82)	0.83 (0.82-0.84)	0.80 (0.79-0.81)	0.81 (0.79-0.82)	0.0000
Occupational Therapist assessment within 72 hours	0.79 (0.77-0.80)	0.82 (0.79-0.84)	0.81 (0.79-0.83)	0.80 (0.77-0.84)	0.0993	0.74 (0.73-0.74)	0.75 (0.74-0.76)	0.71 (0.70-0.72)	0.72 (0.71-0.74)	0.0000
Swallow assessment by a SLT within 72 hours	0.92 (0.90-0.93)	0.94 (0.92-0.96)	0.93 (0.91-0.95)	0.93 (0.89-0.96)	0.3473	0.80 (0.79-0.80)	0.81 (0.80-0.82)	0.79 (0.78-0.81)	0.80 (0.79-0.82)	0.1258
Communication assessment by a SLT within 72 hours	0.53 (0.51-0.55)	0.57 (0.55-0.60)	0.54 (0.52-0.57)	0.50 (0.46-0.55)	0.0191	0.32 (0.32-0.33)	0.36 (0.35-0.37)	0.34 (0.33-0.35)	0.34 (0.32-0.35)	0.0000

Physiotherapist assessment within 24 hours	0.56 (0.55-0.58)	0.47 (0.44-0.50)	0.65 (0.63-0.68)	0.49 (0.45-0.53)	0.0000	0.54 (0.54-0.55)	0.40 (0.40-0.41)	0.52 (0.51-0.53)	0.35 (0.33-0.36)	0.0000
Occupational Therapist assessment within 24 hours	0.49 (0.47-0.50)	0.42 (0.39-0.45)	0.58 (0.56-0.61)	0.43 (0.39-0.47)	0.0000	0.43 (0.42-0.43)	0.30 (0.30-0.31)	0.41 (0.40-0.42)	0.26 (0.25-0.27)	0.0000
Communication assessment by a SLT within 24 hours	0.29 (0.28-0.31)	0.23 (0.20-0.25)	0.39 (0.36-0.41)	0.22 (0.19-0.25)	0.0000	0.17 (0.17-0.17)	0.10 (0.09-0.10)	0.19 (0.18-0.20)	0.08 (0.07-0.09)	0.0000
Outcome measures										
Mortality at three days	0.02 (0.02-0.03)	0.04 (0.03-0.05)	0.03 (0.02-0.03)	0.02 (0.01-0.03)	0.0547	0.04 (0.04-0.04)	0.04 (0.04-0.04)	0.06 (0.05-0.06)	0.06 (0.05-0.06)	0.0000
mRS score 3-6	0.55 (0.53-0.56)	0.58 (0.55-0.60)	0.52 (0.50-0.55)	0.54 (0.50-0.58)	0.0553	0.46 (0.46-0.47)	0.50 (0.49-0.51)	0.54 (0.53-0.55)	0.54 (0.53-0.56)	0.0000
mRS score 3-5*	0.48 (0.46-0.49)	0.51 (0.48-0.54)	0.46 (0.43-0.49)	0.47 (0.43-0.52)	0.1024	0.38 (0.37-0.38)	0.41 (0.40-0.42)	0.44 (0.43-0.45)	0.44 (0.42-0.46)	0.0000
Length of stay										
Length of stay in HASU (days)	3.0 (2.9-3.1)	3.3 (3.2-3.5)	2.9 (2.7-3.0)	3.0 (2.8-3.2)	0.0000					
Length of stay in hospital (days)	7.8 (7.4-8.1)	9.2 (8.5-10.0)	7.6 (7.0-8.2)	8.0 (7.0-9.0)	0.0016	6.6 (6.5-6.6)	7.5 (7.3-7.7)	8.4 (8.2-8.6)	8.7 (8.3-9.1)	0.0000

Note.

Figures are average predicted probabilities (95% confidence intervals) of each measure in each time period controlling for the covariates. SLT = Speech and Language Therapist. mRS = modified Rankin Scale. * Patients who died were not included. †P-value threshold adjusted for multiple testing is 0.0025 for London HASUs and 0.0024 for the rest of England.

Supplementary Table 2A. Quality of care and outcomes across four periods in the week (p-values comparision between Wald test and Likelihood-ratio test)

	London HASUs						Rest of England					
	**Weekday**	**Weekend**	**Weekday**	**Weekend**	**p-value†**	**p-value†**	**Weekday**	**Weekend**	**Weekday**	**Weekend**	**p-value†**	**p-value†**
	**08:00-19:59**	**08:00-19:59**	**20:00-07:59**	**20:00-07:59**	**Wald**	**Likelihood-ratio test**	**08:00-19:59**	**08:00-19:59**	**20:00-07:59**	**20:00-07:59**	**Wald**	**Likelihood-ratio test**
Quality of care measures that do not vary across the week in London HASUs												
Brain scan within one hour	0.60 (0.58-0.61)	0.61 (0.58-0.63)	0.63 (0.60-0.65)	0.65 (0.61-0.68)	0.0344	0.0336	0.44 (0.44-0.45)	0.41 (0.40-0.41)	0.40 (0.39-0.40)	0.39 (0.38-0.41)	0.0000	0.0000
Brain scan within 12 hours	0.97 (0.96-0.97)	0.96 (0.95-0.97)	0.95 (0.94-0.96)	0.95 (0.93-0.96)	0.0093	0.0110	0.90 (0.90-0.90)	0.88 (0.87-0.89)	0.84 (0.83-0.84)	0.83 (0.82-0.84)	0.0000	0.0000
Dysphagia screen within four hours	0.74 (0.72-0.75)	0.75 (0.73-0.77)	0.77 (0.75-0.79)	0.79 (0.76-0.82)	0.0029	0.0026	0.70 (0.70-0.71)	0.65 (0.64-0.66)	0.60 (0.59-0.61)	0.58 (0.56-0.59)	0.0000	0.0000
Assessment by a nurse trained in stroke management within 24 hours	0.96 (0.95-0.96)	0.94 (0.93-0.96)	0.95 (0.94-0.96)	0.95 (0.94-0.97)	0.1872	0.1896	0.89 (0.88-0.89)	0.85 (0.85-0.86)	0.86 (0.86-0.87)	0.83 (0.82-0.84)	0.0000	0.0000
Administration of intravenous thrombolysis to eligible patients	0.88 (0.86-0.90)	0.88 (0.84-0.92)	0.86 (0.82-0.91)	0.88 (0.82-0.95)	0.9327	0.9341	0.81 (0.80-0.82)	0.80 (0.78-0.82)	0.76 (0.74-0.78)	0.76 (0.72-0.79)	0.0000	0.0000
Door-to-needle time within one hour in patients receiving thrombolysis	0.84 (0.81-0.87)	0.89 (0.85-0.93)	0.79 (0.74-0.84)	0.84 (0.77-0.91)	0.0269	0.0233	0.60 (0.59-0.62)	0.48 (0.45-0.50)	0.38 (0.35-0.40)	0.37 (0.33-0.41)	0.0000	0.0000
Quality of care: measures that vary significantly across the week												
Assessment by a stroke specialist consultant physician	0.39 (0.38-.40)	0.30 (0.27-0.32)	0.63 (0.61-0.66)	0.64 (0.60-0.68)	0.0000	0.0000	0.48 (0.48-0.49)	0.30 (0.29-0.31)	0.51 (0.51-0.52)	0.42 (0.41-0.44)	0.0000	0.0000

within 12 hours												
Assessment by a stroke specialist consultant physician within 24 hours	0.90 (0.89-0.91)	0.87 (0.85-0.89)	0.90 (0.88-0.91)	0.92 (0.90-0.94)	0.0043	0.0048	0.80 (0.79-0.80)	0.65 (0.65-0.66)	0.75 (0.74-0.75)	0.62 (0.61-0.64)	0.0000	0.0000
Admission to a stroke unit within four hours	0.62 (0.60-0.63)	0.64 (0.61-0.66)	0.67 (0.65-0.70)	0.70 (0.67-0.74)	0.0000	0.0000	0.63 (0.63-0.63)	0.59 (0.58-0.60)	0.55 (0.54-0.56)	0.53 (0.52-0.55)	0.0000	0.0000
Physiotherapist assessment within 72 hours	0.83 (0.82-0.84)	0.86 (0.84-0.88)	0.85 (0.83-0.87)	0.84 (0.81-0.87)	0.0693	0.0666	0.82 (0.81-0.82)	0.83 (0.82-0.84)	0.81 (0.81-0.82)	0.82 (0.80-0.83)	0.0010	0.0009
Occupational Therapist assessment within 72 hours	0.79 (0.78-0.80)	0.82 (0.80-0.84)	0.81 (0.79-0.82)	0.80 (0.76-0.83)	0.0967	0.0936	0.73 (0.73-0.74)	0.75 (0.75-0.76)	0.73 (0.72-0.74)	0.73 (0.72-0.74)	0.0000	0.0000
Swallow assessment by a SLT within 72 hours	0.92 (0.91-0.93)	0.93 (0.91-0.95)	0.93 (0.91-0.95)	0.91 (0.88-0.95)	0.5838	0.5795	0.80 (0.80-0.81)	0.81 (0.80-0.82)	0.79 (0.78-0.80)	0.80 (0.78-0.82)	0.0946	0.0946
Communication assessment by a SLT within 72 hours	0.53 (0.51-0.54)	0.56 (0.54-0.59)	0.55 (0.53-0.58)	0.52 (0.48-0.56)	0.0739	0.0735	0.33 (0.32-0.33)	0.36 (0.35-0.37)	0.34 (0.33-0.35)	0.34 (0.32-0.35)	0.0000	0.0000
Physiotherapist assessment within 24 hours	0.56 (0.54-0.57)	0.47 (0.45-0.50)	0.65 (0.63-0.68)	0.48 (0.44-0.52)	0.0000	0.0000	0.54 (0.54-0.55)	0.41 (0.40-0.41)	0.53 (0.52-0.54)	0.35 (0.34-0.37)	0.0000	0.0000
Occupational Therapist assessment within 24 hours	0.49 (0.47-0.50)	0.42 (0.40-0.45)	0.58 (0.55-0.60)	0.41 (0.37-0.45)	0.0000	0.0000	0.43 (0.42-0.43)	0.31 (0.30-0.31)	0.42 (0.42-0.43)	0.26 (0.25-0.27)	0.0000	0.0000
Communication assessment by a SLT within 24 hours	0.29 (0.28-0.31)	0.22 (0.20-0.24)	0.39 (0.37-0.42)	0.23 (0.20-0.27)	0.0000	0.0000	0.17 (0.17-0.17)	0.10 (0.09-0.10)	0.19 (0.18-0.20)	0.08 (0.07-0.09)	0.0000	0.0000
Outcome measures												
Mortality at three days	0.03 (0.02-0.03)	0.03 (0.02-0.04)	0.03 (0.02-0.04)	0.02 (0.01-0.03)	0.3310	0.3298	0.04 (0.04-0.05)	0.04 (0.04-0.04)	0.05 (0.04-0.05)	0.05 (0.04-0.05)	0.1055	0.1030
mRS score 3-6	0.55 (0.53-	0.55 (0.52-	0.55 (0.52-	0.56 (0.53-	0.8672	0.8673	0.48 (0.48-	0.49 (0.48-	0.51 (0.50-	0.51 (0.50-	0.0000	0.0000

	0.56)	0.57)	0.57)	0.59)			0.48)	0.50)	0.51)	0.52)		
mRS score 3-5*	0.49 (0.47-0.50)	0.47 (0.45-0.50)	0.48 (0.45-0.50)	0.48 (0.44-0.51)	0.7497	0.7494	0.40 (0.39-0.40)	0.40 (0.39-0.41)	0.40 (0.39-0.41)	0.40 (0.39-0.41)	0.3746	0.3750
Length of stay												
Length of stay in HASU (days)	3.1 (3.0-3.2)	3.4 (3.2-3.5)	3.0 (2.9-3.1)	3.1 (2.9-3.3.)	0.0007	0.0008						
Length of stay in hospital (days)	10.8 (10.2-11.3)	12.1 (11.1-13.1)	10.8 (10.0-11.7)	11.5 (10.2-12.9)	0.0359	0.0359	8.5 (8.4-8.6)	9.2 (9.0-9.4)	9.7(9.4-9.9)	10.1 (9.6-10.5)	0.0000	0.0000

Note.

Figures are average predicted probabilities (95% confidence intervals) of each measure in each time period controlling for the covariates. SLT = Speech and Language Therapist. mRS = modified Rankin Scale. * Patients who died were not included. †P-value threshold adjusted for multiple testing is 0.0025 for London HASUs and 0.0024 for the rest of England.

Supplementary Table 3. Quality of care and outcomes across four periods in the week cotrolling for NIHSS score on arrival

	**London HASUs**					**Rest of England**				
	**Weekday**	**Weekend**	**Weekday**	**Weekend**	**†p-value**	**Weekday**	**Weekend**	**Weekday**	**Weekend**	**†p-value**
	**08:00-19:59**	**08:00-19:59**	**20:00-07:59**	**20:00-07:59**		**08:00-19:59**	**08:00-19:59**	**20:00-07:59**	**20:00-07:59**	
Quality of care measures that do not vary across the week in London HASUs										
Brain scan within one hour	0.60 (0.59-0.62)	0.61 (0.59-0.64)	0.63 (0.61-0.66)	0.65 (0.62-0.69)	0.0256	0.47 (0.46-0.47)	0.43 (0.42-0.44)	0.41 (0.41-0.42)	0.41 (0.40-0.43)	0.0000
Brain scan within 12 hours	0.97 (0.97-0.98)	0.97 (0.96-0.98)	0.95 (0.94-0.96)	0.94 (0.92-0.96)	0.0012	0.91 (0.91-0.92)	0.89 (0.89-0.90)	0.85 (0.84-0.86)	0.85 (0.84-0.86)	0.0000
Dysphagia screen within four hours	0.74 (0.73-0.76)	0.76 (0.74-0.79)	0.78 (0.76-0.81)	0.80 (0.77-0.84)	0.0003	0.74 (0.73-0.74)	0.68 (0.68-0.69)	0.64 (0.63-0.65)	0.61 (0.60-0.63)	0.0000
Assessment by a nurse trained in stroke management within 24 hours	0.96 (0.96-0.97)	0.95 (0.94-0.96)	0.95 (0.94-0.96)	0.96 (0.95-0.98)	0.1191	0.92 (0.92-0.93)	0.89 (0.89-0.90)	0.90 (0.90-0.91)	0.88 (0.87-0.89)	0.0000
Administration of intravenous thrombolysis to eligible patients	0.89 (0.86-0.91)	0.89 (0.85-0.93)	0.88 (0.83-0.92)	0.87 (0.81-0.94)	0.9436	0.83 (0.82-0.84)	0.83 (0.81-0.84)	0.78 (0.76-0.80)	0.79 (0.75-0.82)	0.0000
Door-to-needle time within one hour in patients receiving thrombolysis	0.84 (0.81-0.87)	0.89 (0.85-0.93)	0.79 (0.74-0.85)	0.85 (0.78-0.92)	0.0673	0.62 (0.60-0.63)	0.48 (0.46-0.51)	0.39 (0.36-0.42)	0.38 (0.33-0.42)	0.0000
Quality of care: measures that vary significantly across the week										
Assessment by a stroke specialist consultant physician within 12 hours	0.39 (0.38-.40)	0.29 (0.27-0.31)	0.64 (0.62-0.67)	0.65 (0.62-0.69)	0.0000	0.52 (0.52-0.53)	0.33 (0.32-0.34)	0.55 (0.54-0.56)	0.46 (0.44-0.47)	0.0000
Assessment by a stroke specialist consultant physician within 24 hours	0.90 (0.89-0.91)	0.88 (0.86-0.89)	0.90 (0.89-0.92)	0.94 (0.92-0.96)	0.0005	0.84 (0.83-0.84)	0.70 (0.69-0.71)	0.79 (0.78-0.80)	0.67 (0.65-0.68)	0.0000
Admission to a stroke unit within four hours	0.62 (0.61-0.64)	0.65 (0.62-0.68)	0.69 (0.66-0.71)	0.71 (0.67-0.74)	0.0000	0.67 (0.67-0.68)	0.63 (0.62-0.64)	0.59 (0.58-0.60)	0.57 (0.56-0.59)	0.0000
Physiotherapist assessment within 72 hours	0.84 (0.83-0.85)	0.87 (0.85-0.89)	0.86 (0.84-0.88)	0.85 (0.82-0.88)	0.1845	0.85 (0.85-0.85)	0.86 (0.86-0.87)	0.85 (0.84-0.85)	0.85 (0.84-0.86)	0.0022
Occupational Therapist assessment within 72 hours	0.80 (0.79-0.81)	0.83 (0.81-0.85)	0.82 (0.80-0.84)	0.81 (0.78-0.84)	0.0707	0.77 (0.77-0.77)	0.79 (0.78-0.80)	0.77 (0.76-0.78)	0.77 (0.76-0.78)	0.0005
Swallow assessment by a SLT within 72 hours	0.93 (0.91-0.94)	0.95 (0.93-0.97)	0.94 (0.92-0.96)	0.91 (0.87-0.85)	0.2298	0.83 (0.82-0.83)	0.84 (0.83-0.85)	0.82 (0.81-0.83)	0.82 (0.80-0.84)	0.1677

Communication assessment by a SLT within 72 hours	0.54 (0.52-0.55)	0.57 (0.54-0.60)	0.56 (0.54-0.59)	0.53 (0.49-0.57)	0.1069	0.33 (0.32-0.33)	0.36 (0.35-0.37)	0.33 (0.32-0.34)	0.34 (0.32-0.36)	0.0000
Physiotherapist assessment within 24 hours	0.57 (0.55-0.58)	0.49 (0.46-0.51)	0.66 (0.64-0.69)	0.49 (0.45-0.53)	0.0000	0.57 (0.57-0.58)	0.43 (0.42-0.44)	0.57 (0.56-0.58)	0.38 (0.36-0.39)	0.0000
Occupational Therapist assessment within 24 hours	0.49 (0.48-0.51)	0.43 (0.41-0.46)	0.59 (0.56-0.61)	0.42 (0.38-0.46)	0.0000	0.45 (0.45-0.46)	0.33 (0.32-0.33)	0.46 (0.45-0.47)	0.28 (0.27-0.30)	0.0000
Communication assessment by a SLT within 24 hours	0.30 (0.28-0.31)	0.22 (0.20-0.25)	0.40 (0.38-0.43)	0.24 (0.20-0.27)	0.0000	0.17 (0.17-0.18)	0.10 (0.09-0.10)	0.19 (0.18-0.20)	0.08 (0.07-0.09)	0.0000
Outcome measures										
Mortality at three days	0.02 (0.02-0.03)	0.03 (0.02-0.04)	0.03 (0.02-0.04)	0.02 (0.01-0.03)	0.2987	0.03 (0.03-0.03)	0.03 (0.02-0.03)	0.03 (0.03-0.03)	0.03 (0.02-0.03)	0.6904
mRS score 3-6	0.53 (0.52-0.54)	0.52 (0.50-0.55)	0.53 (0.51-0.55)	0.54 (0.510-0.57)	0.8754	0.43 (0.43-0.44)	0.44 (0.43-0.45)	0.45 (0.44-0.46)	0.46 (0.44-0.47)	0.0000
mRS score 3-5*	0.49 (0.47-0.50)	0.47 (0.45-0.50)	0.48 (0.45-0.50)	0.48 (0.44-0.51)	0.7497	0.40 (0.39-0.40)	0.40 (0.39-0.41)	0.40 (0.39-0.41)	0.40 (0.39-0.41)	0.3746
Length of stay										
Length of stay in HASU (days)	3.1 (3.0-3.2)	3.3 (3.2-3.5)	3.0 (2.9-3.1)	3.1 (2.9-3.3)	0.0080					
Length of stay in hospital (days)	12.8 (12.1-13.6)	14.4 (13.2-15.6)	13.2 (12.2-14.3)	13.2 (11.7-14.7)	0.0562	8.9 (8.7-9.0)	9.5 (9.3-9.8)	9.9 (9.6-10.2)	10.4 (10.0-10.9)	0.0000

Note.

Figures are average predicted probabilities (95% confidence intervals) of each measure in each time period controlling for the covariates. SLT = Speech and Language Therapist. mRS = modified Rankin Scale. * Patients who died were not included. †P-value threshold adjusted for multiple testing is 0.0025 for London HASUs and 0.0024 for the rest of England.

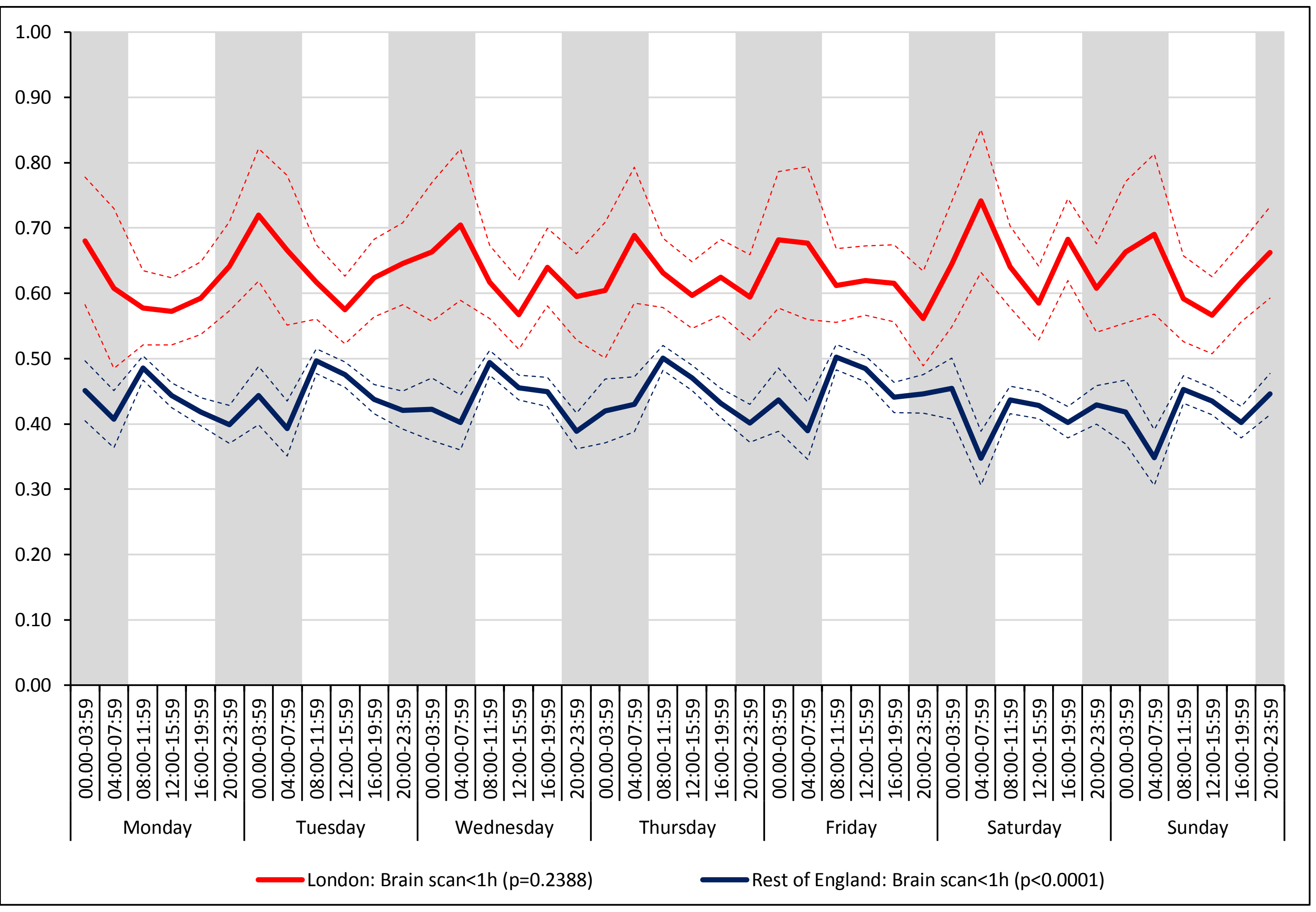

Figure S1(a). Brain scan within one hour

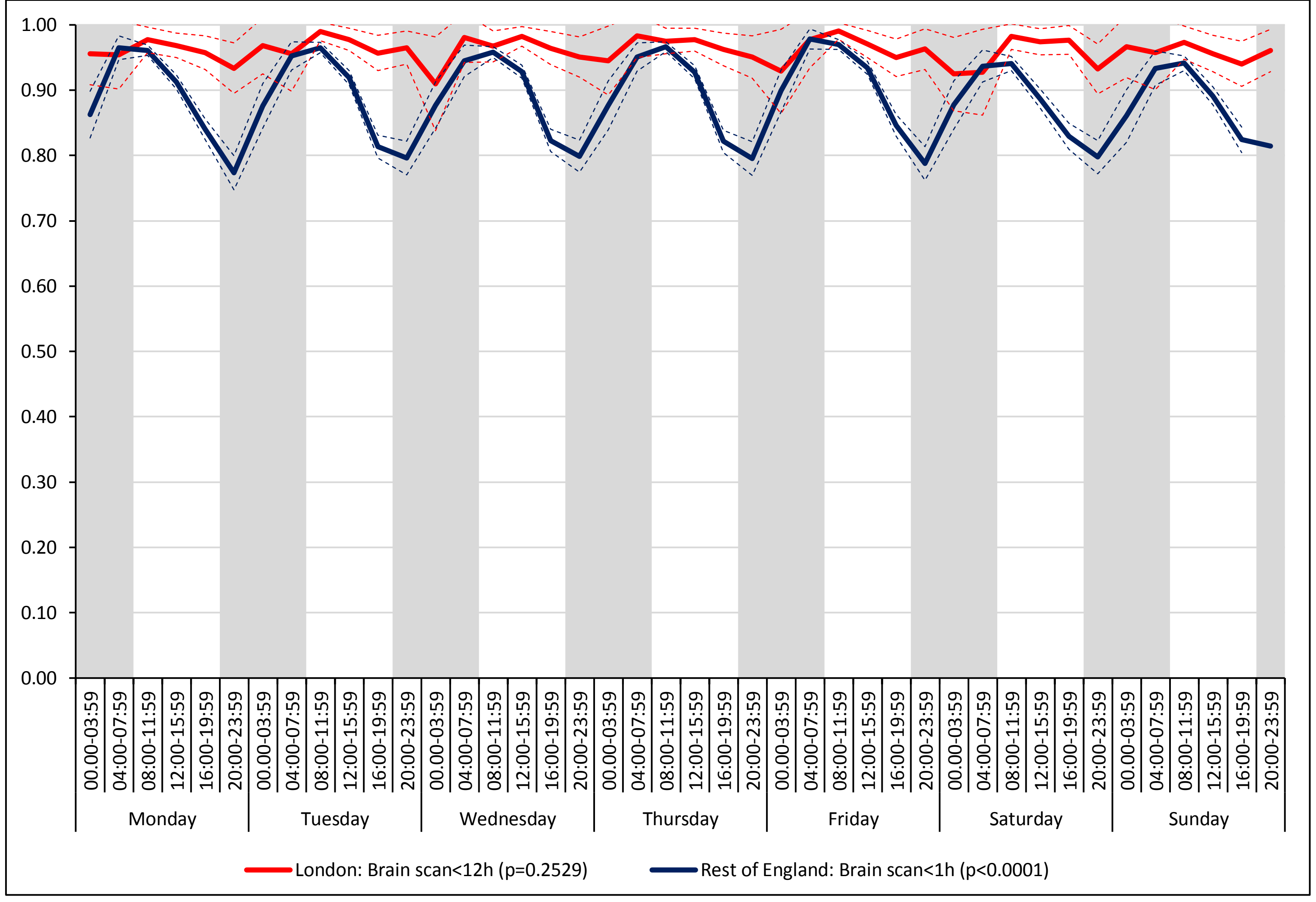

Figure S1(b). Brain scan within 12 hours

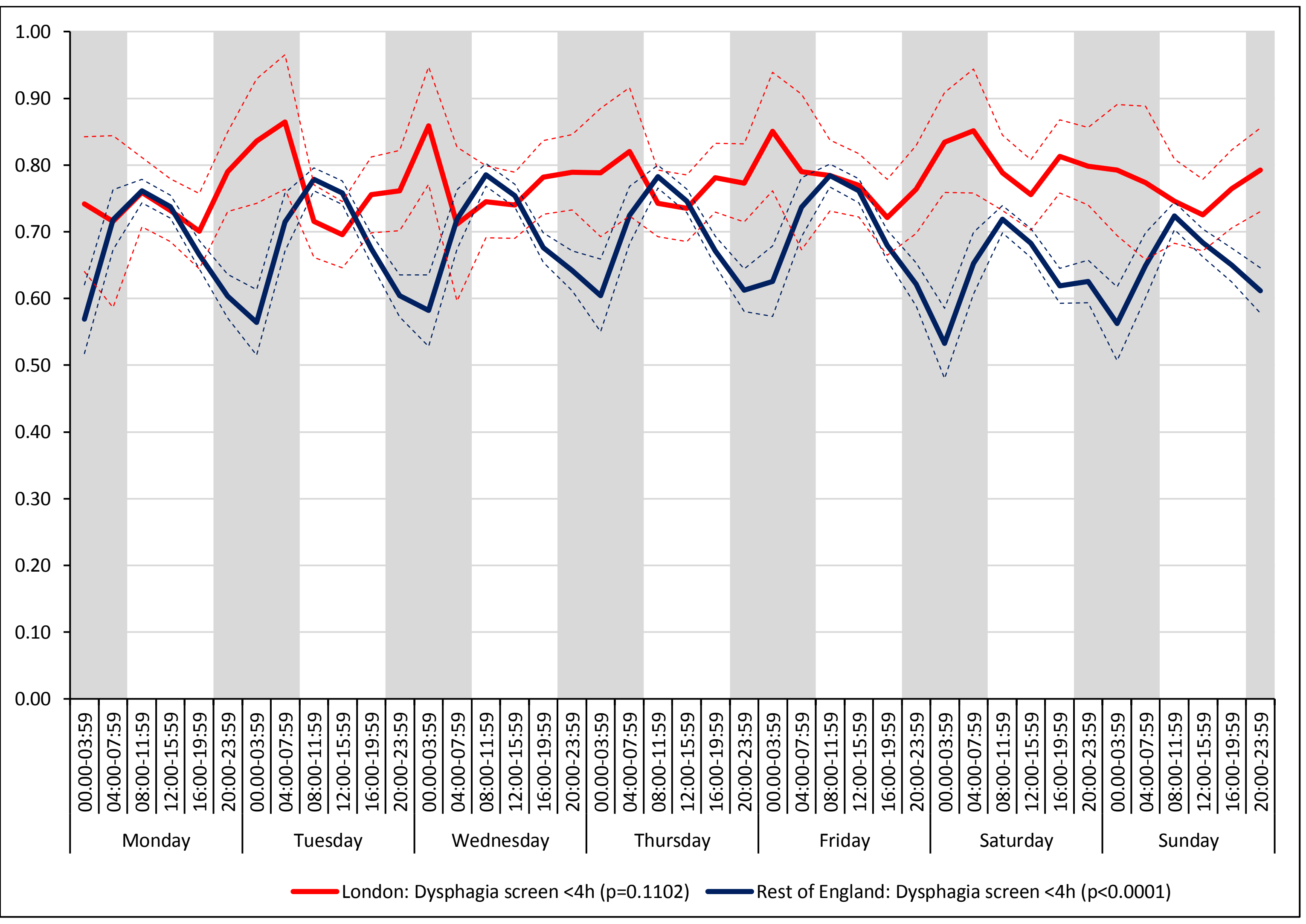

Figure S1(c). Dysphagia screen within four hours

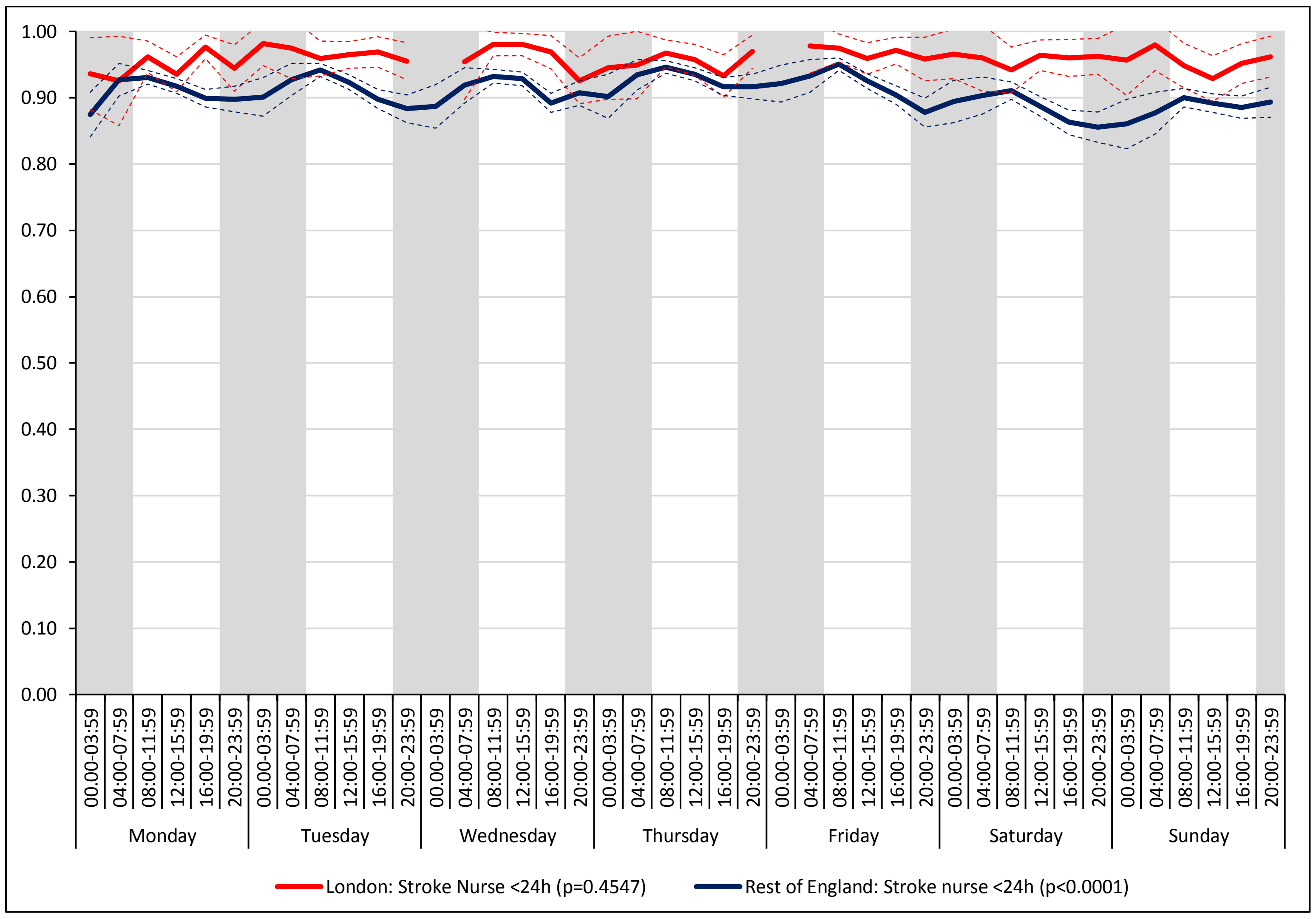

Figure S1(d). Nurse assessment within 24 hours

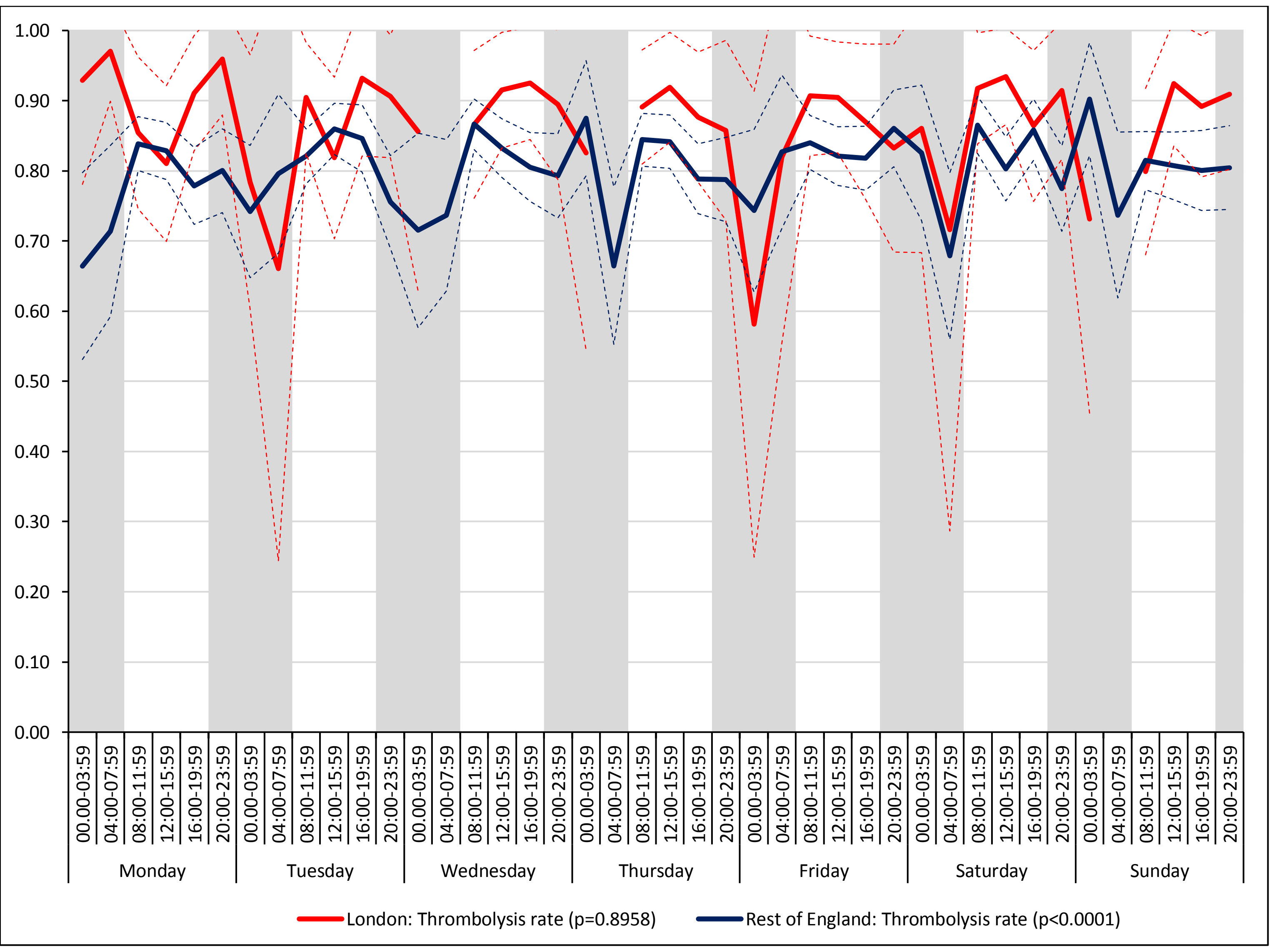

Figure S1(e). Administration of intravenous thrombolysis to eligible patients

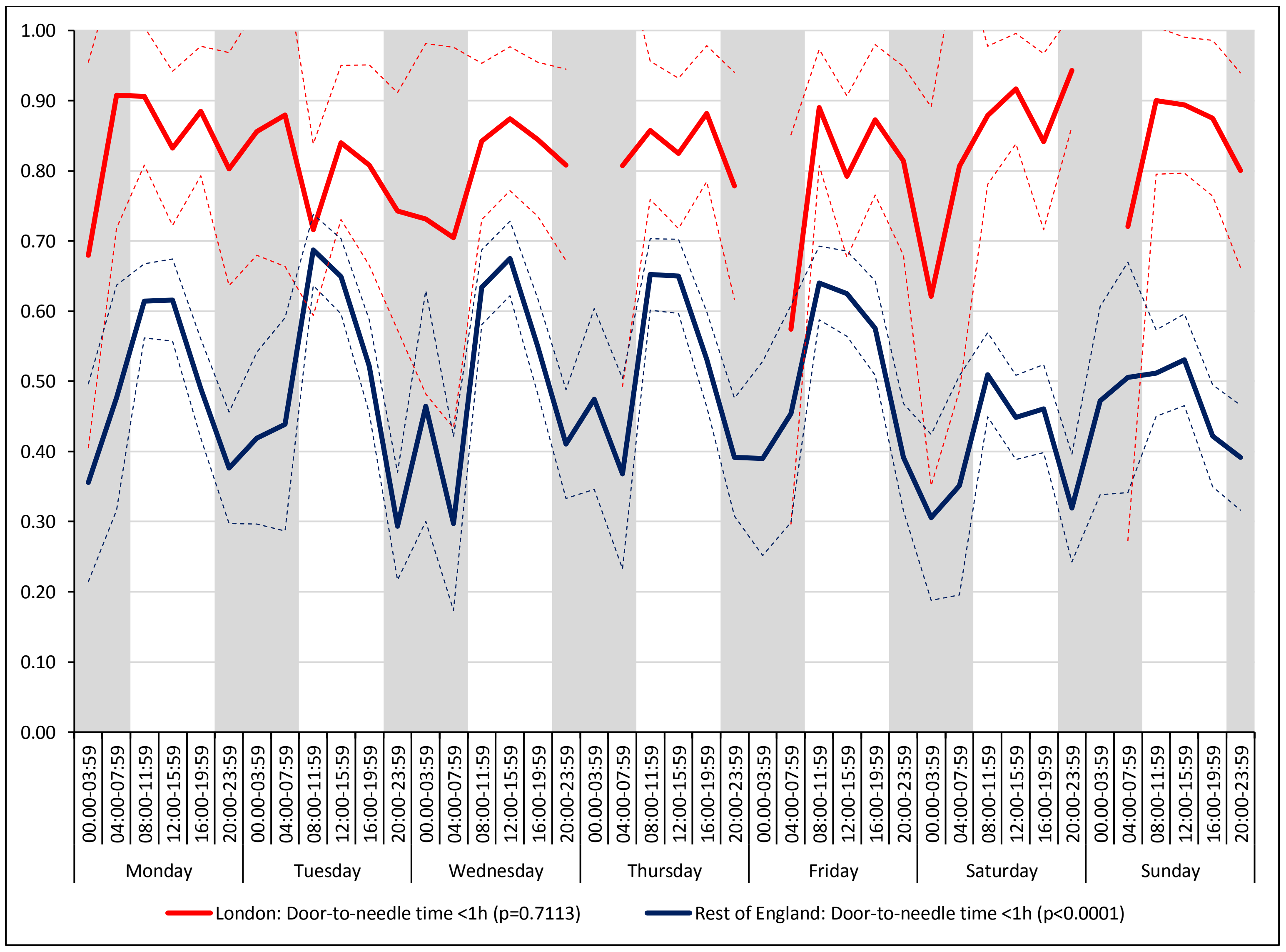

Figure S1(f). Door-to-needle time within one hour

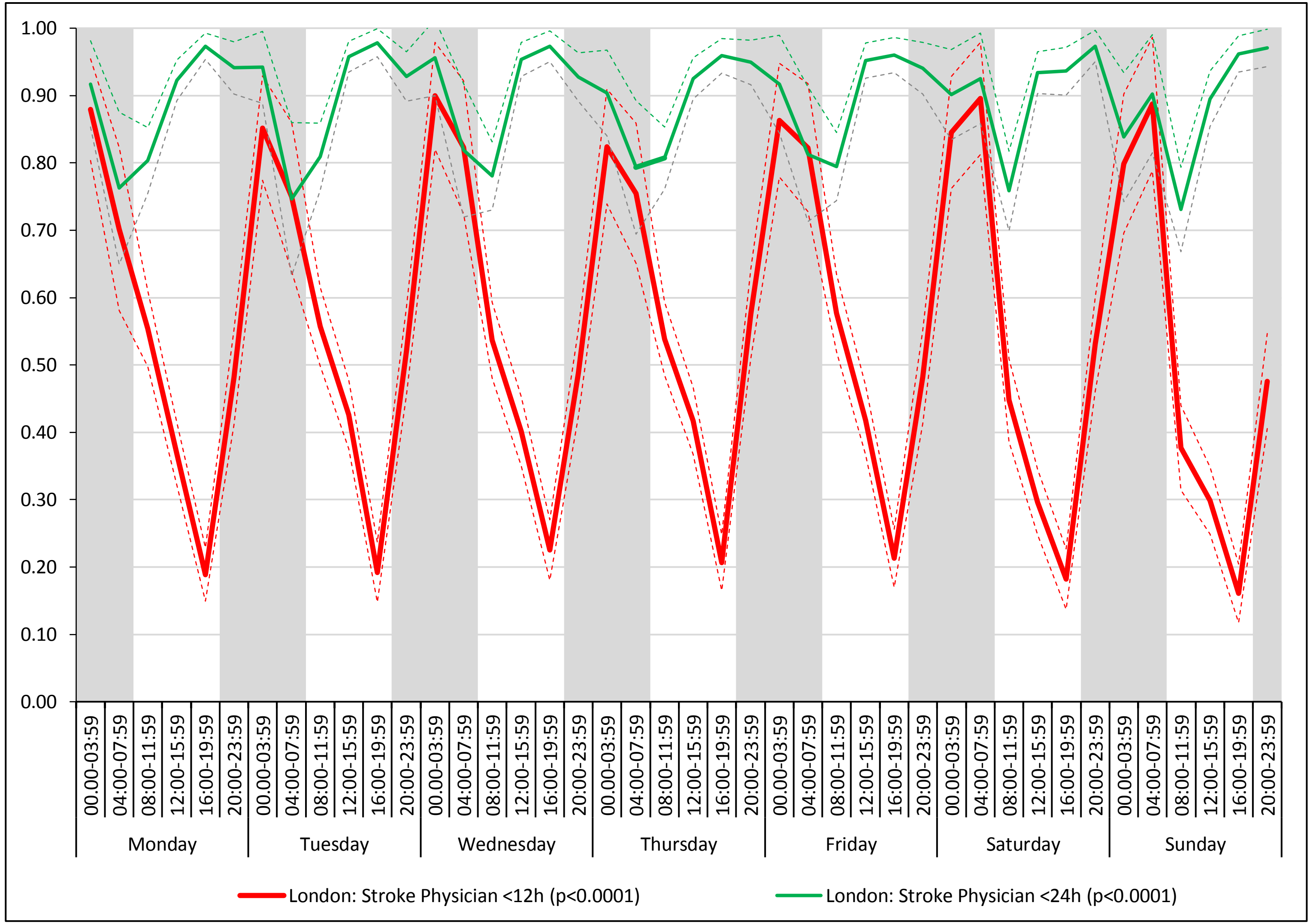

Figure S2(a). Assessment by a stroke consultant in London HASUs

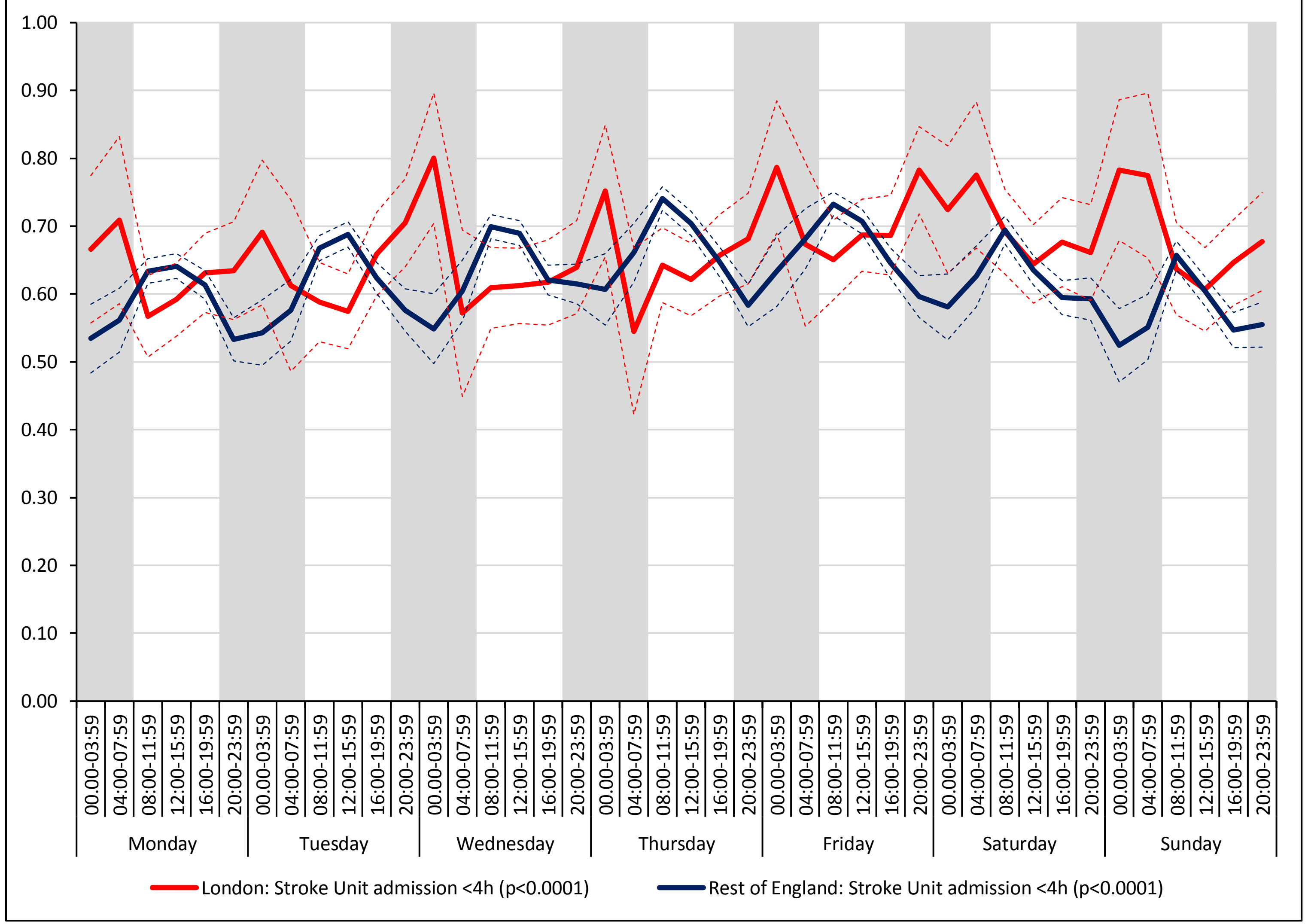

Figure S2(b). Admission to a stroke unit within four hours

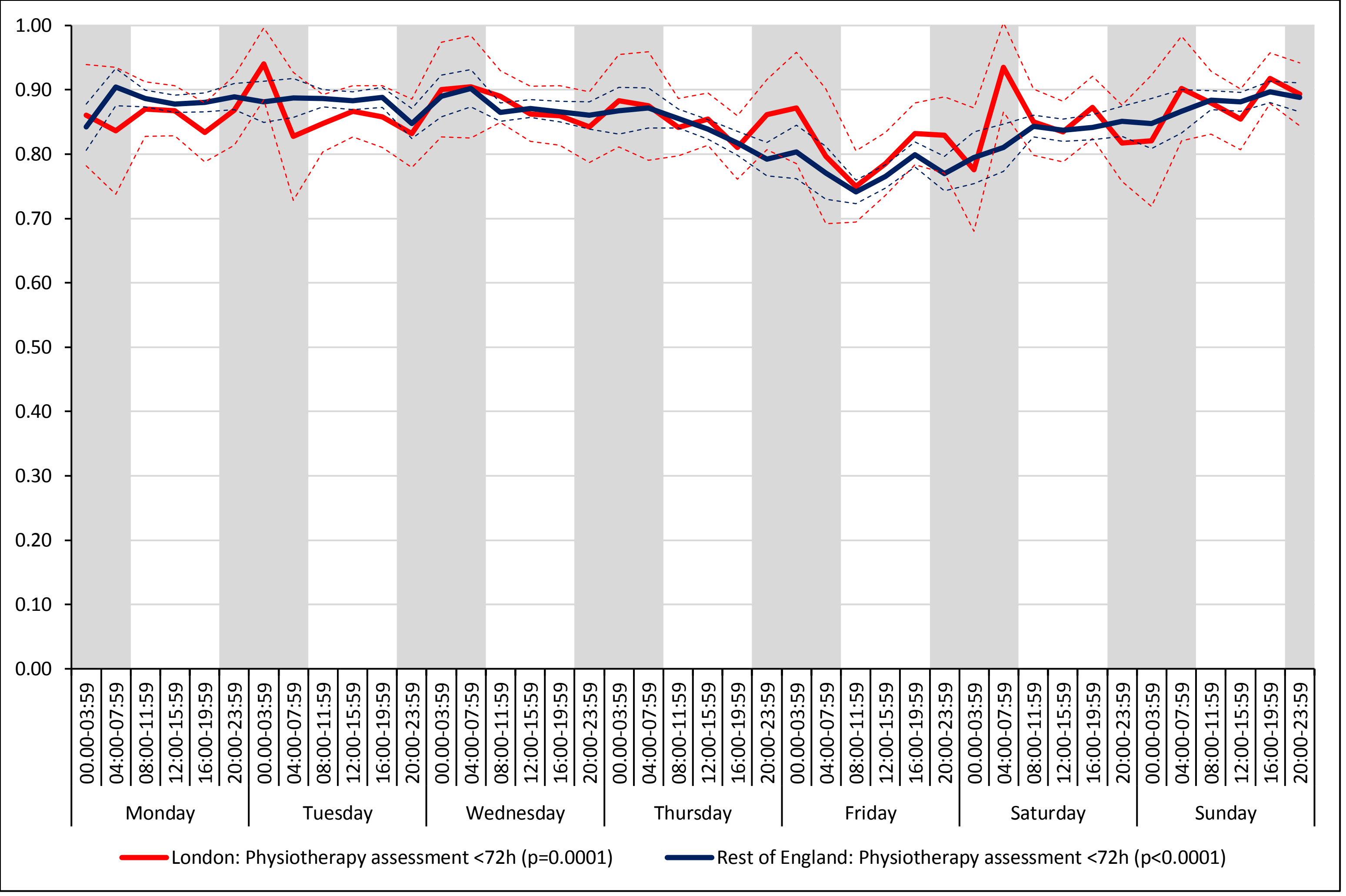

Figure S3(a). Physiotherapist assessment within 72 hours

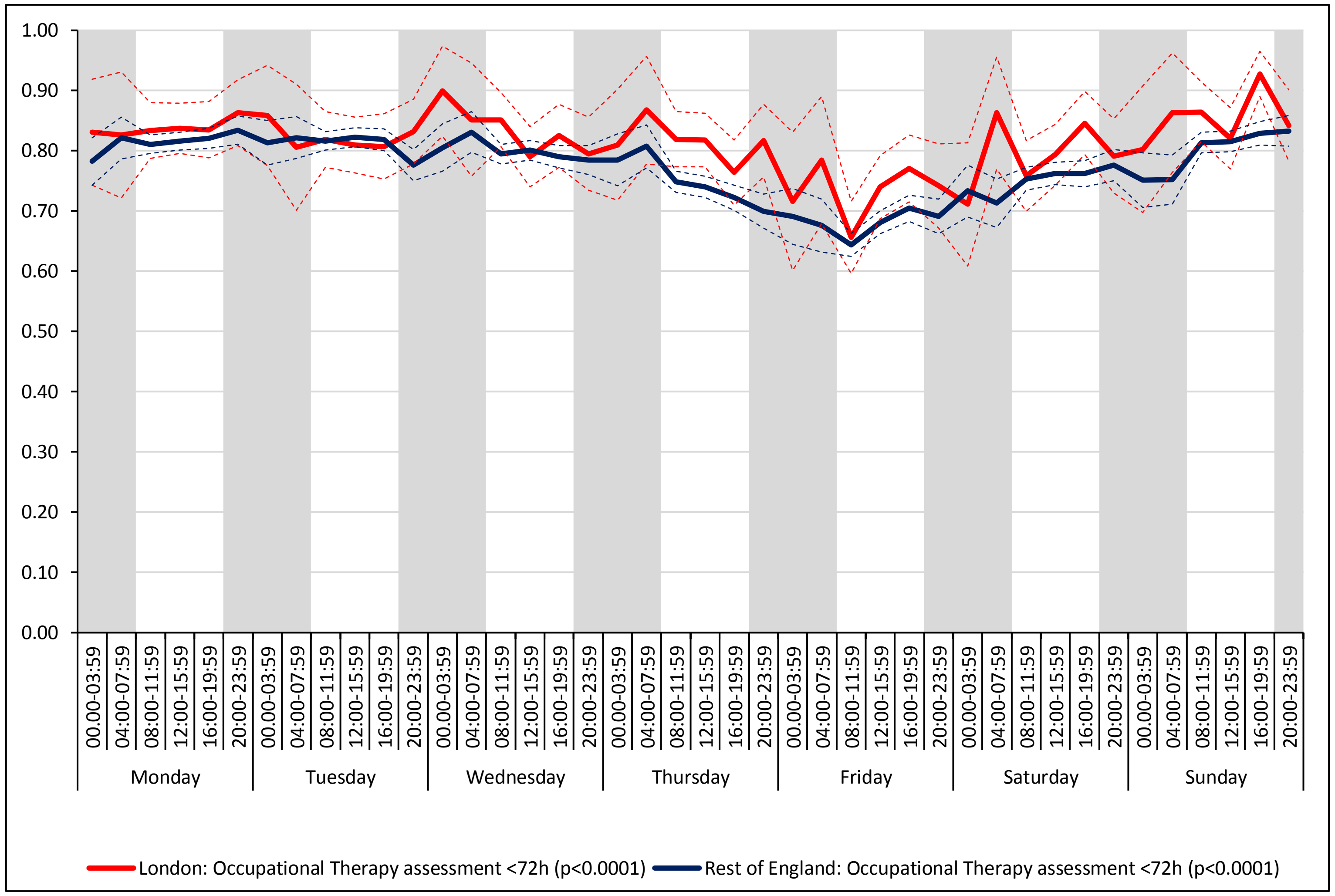

Figure S3(b). Occupational Therapist assessment within 72 hours

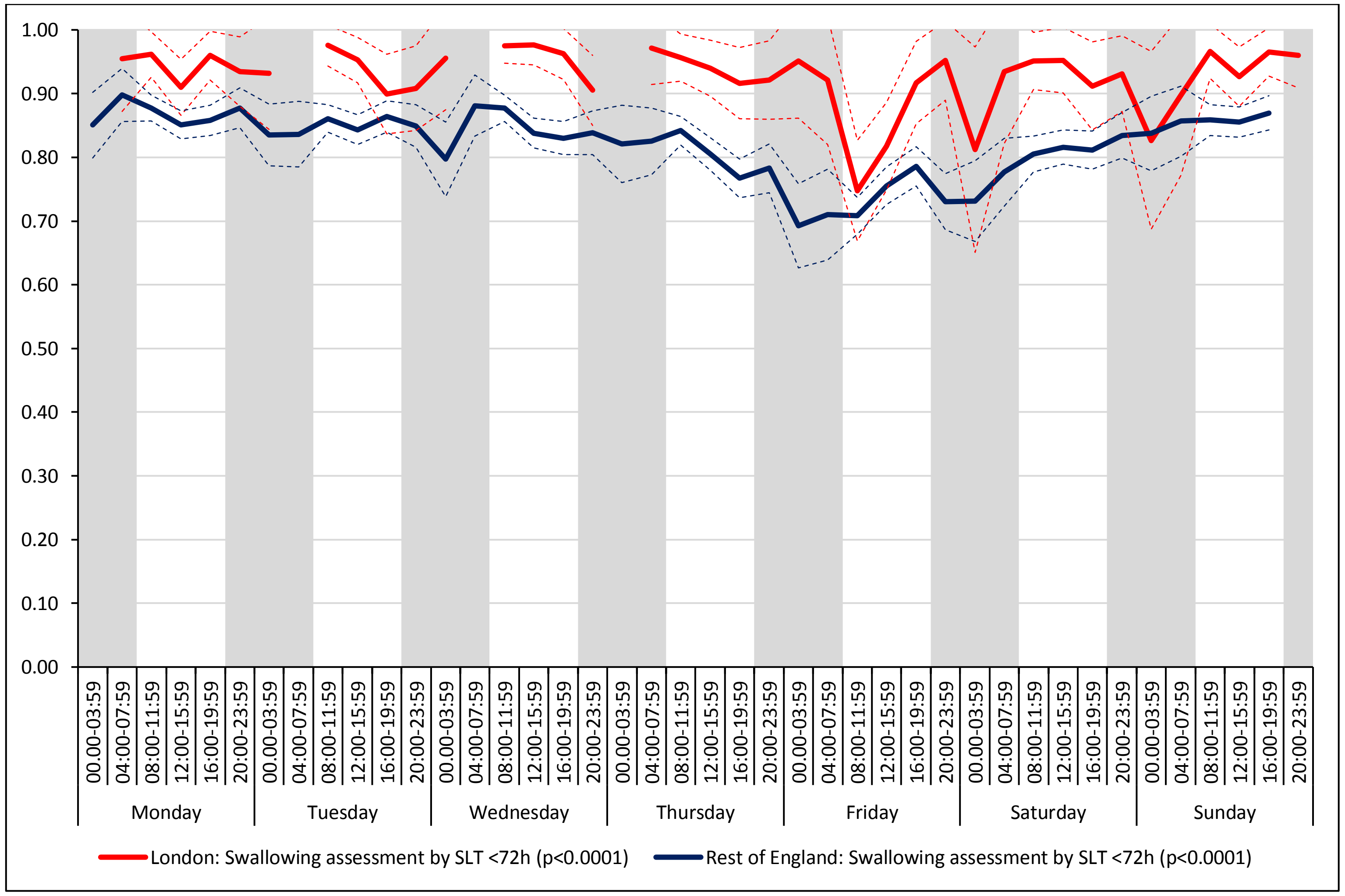

Figure S3(c). Swallow assessment by a SaLT within 72 hours

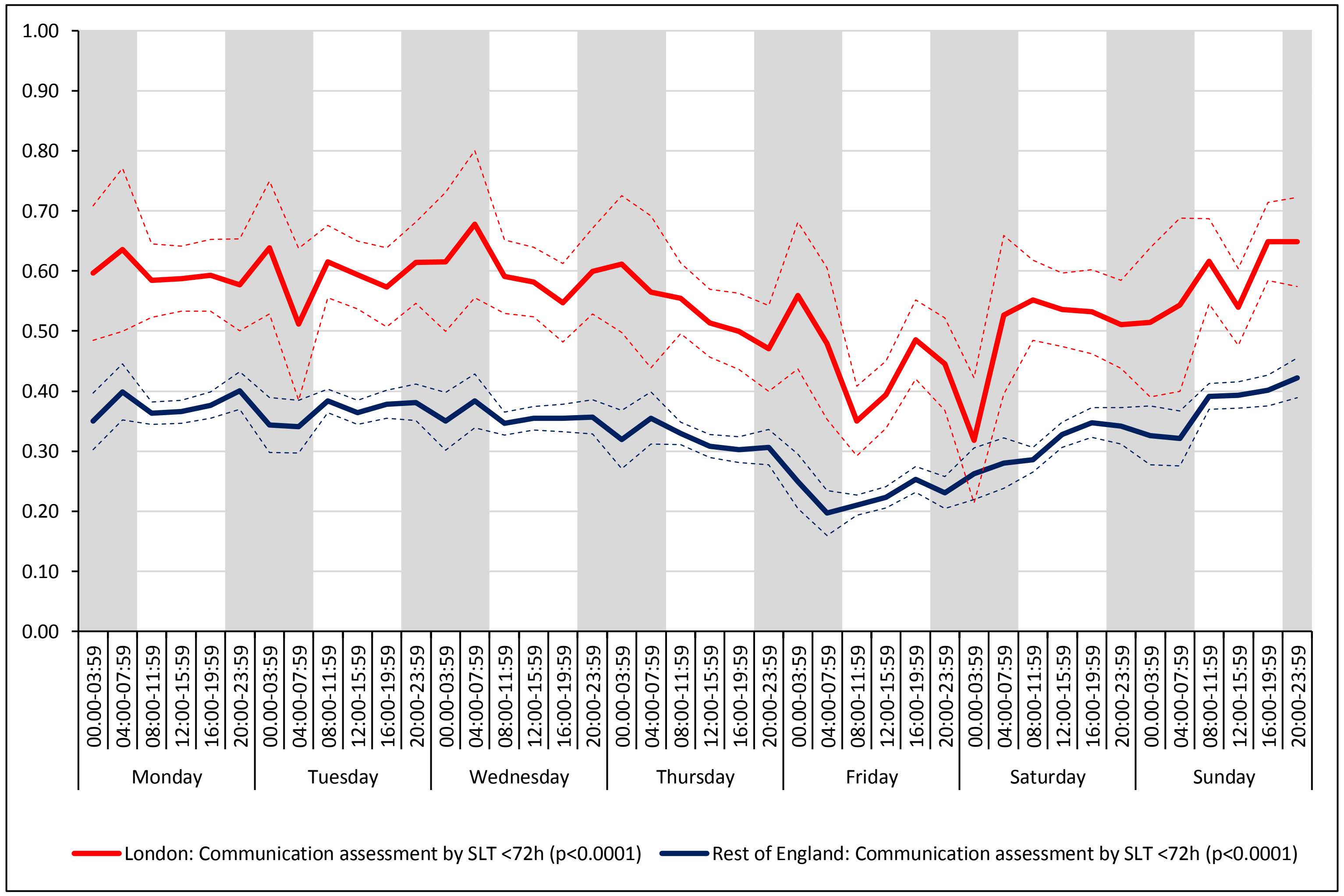

Figure S3(d). Communication assessment by a SaLT within 72 hours

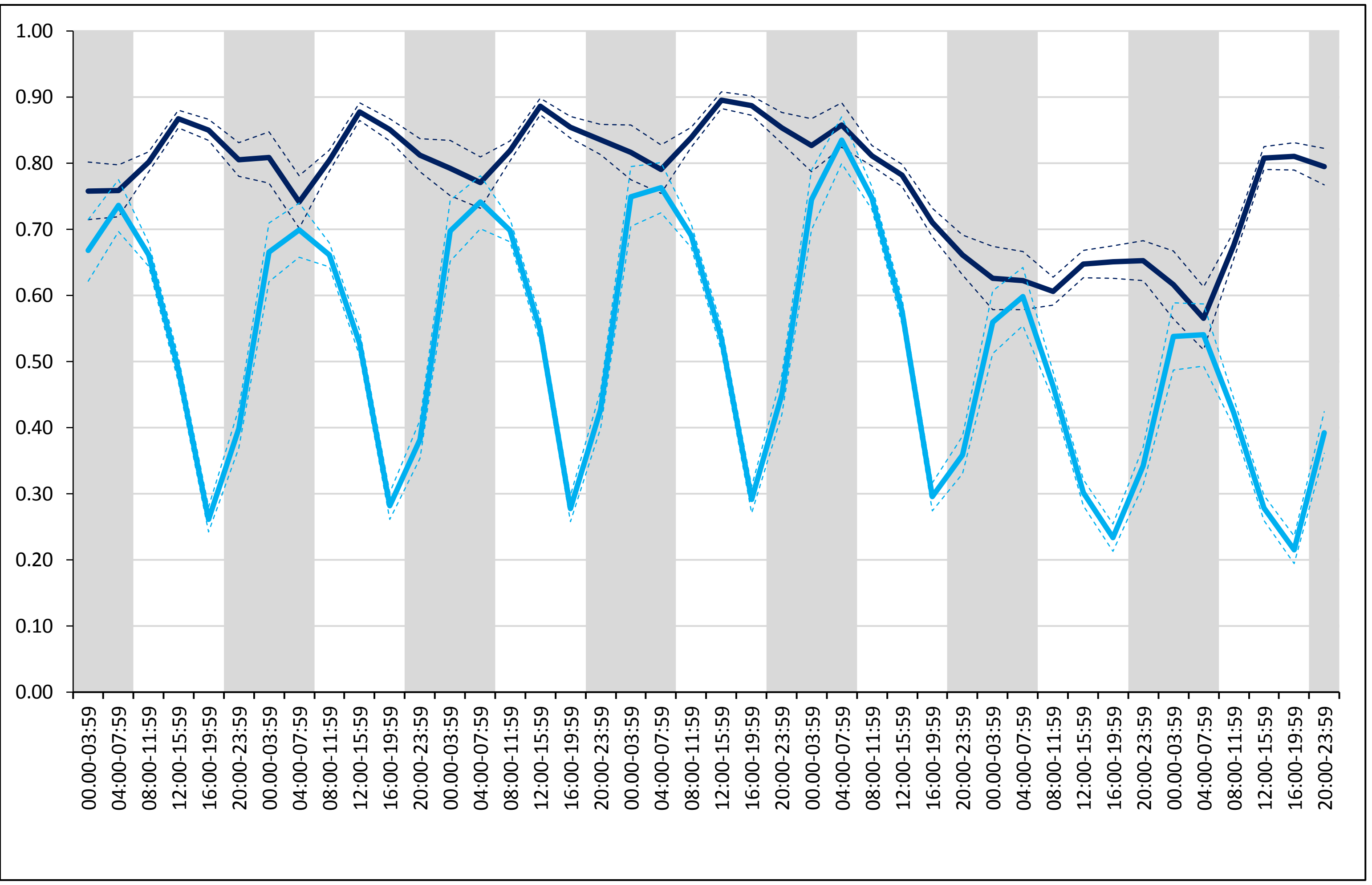

Figure S4(a). Assessment by a stroke consultant in Rest of England

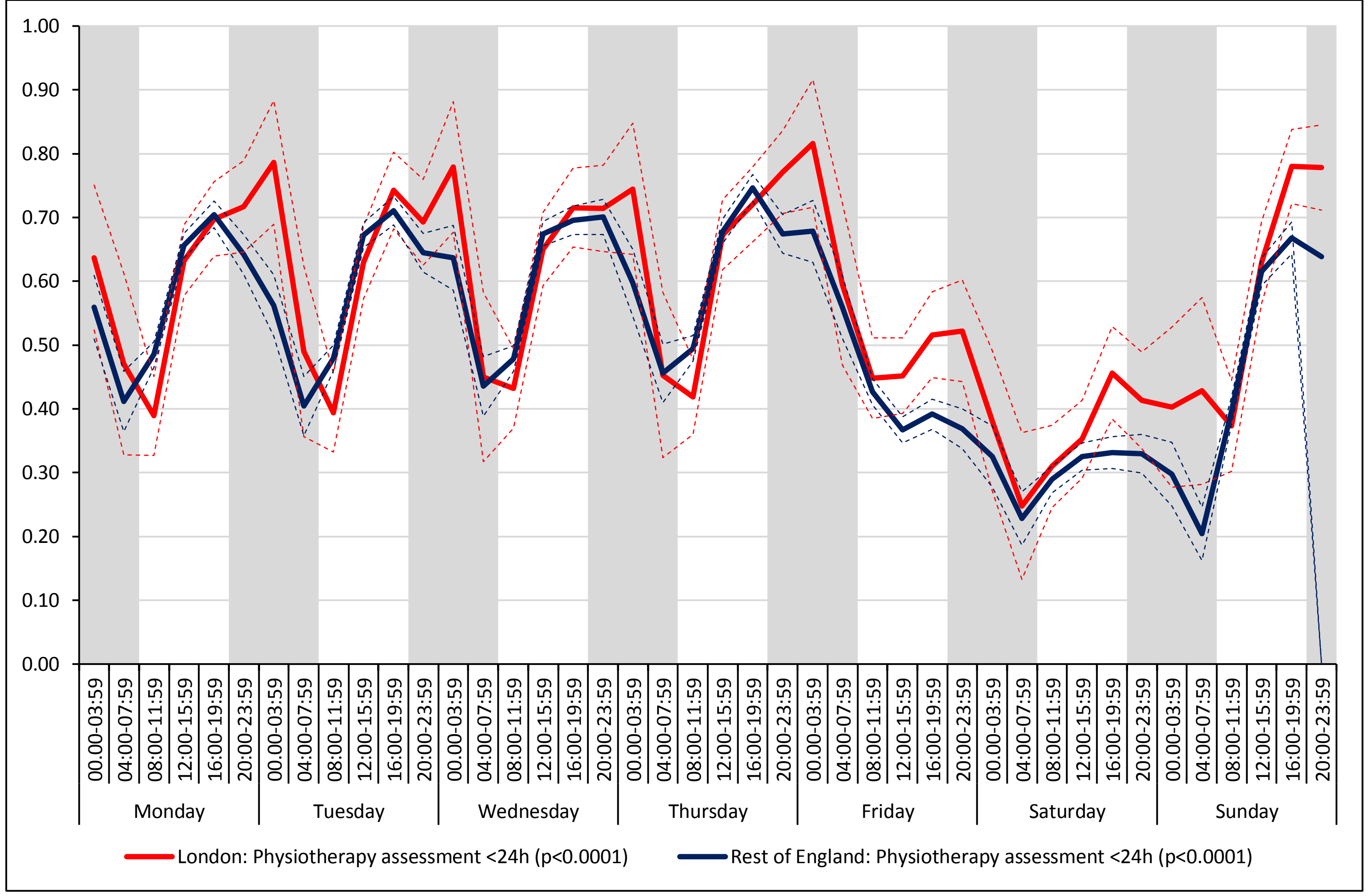

Figure S4(b). Physiotherapist assessment within 24 hours

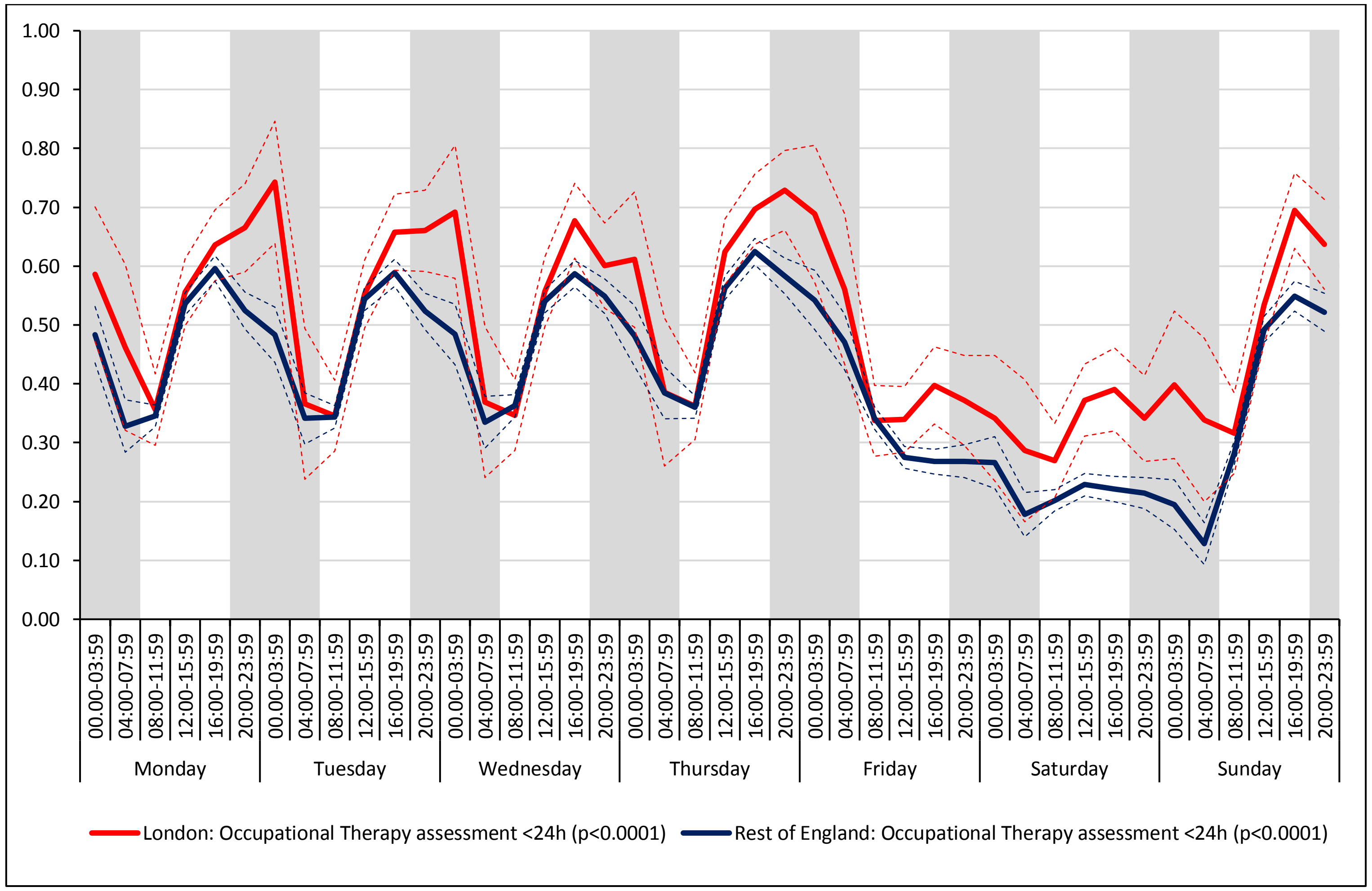

Figure S4(c). Occupational Therapist assessment within 24 hours

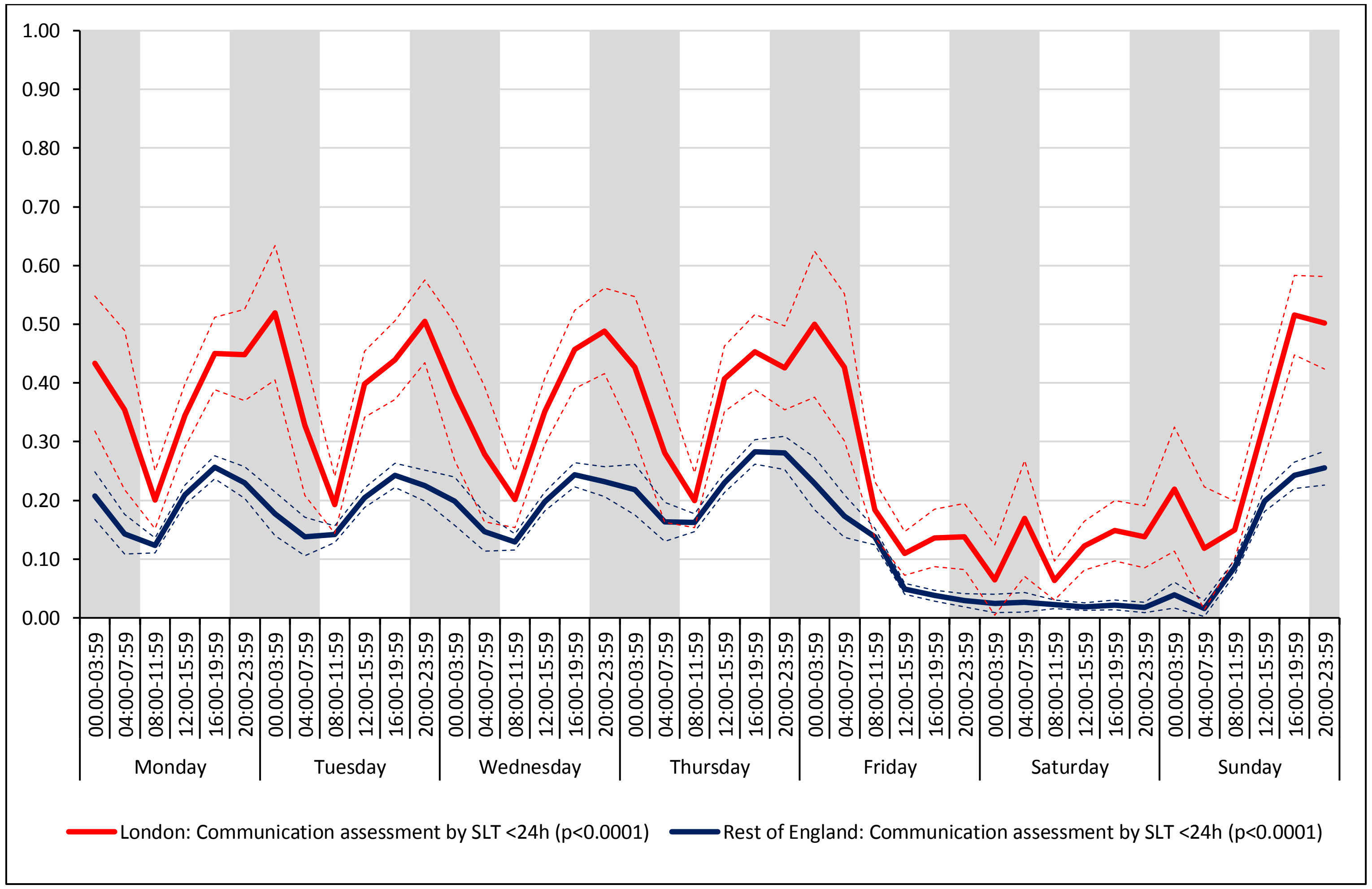

Figure S4(d). Communication assessment by a SaLT within 24 hours

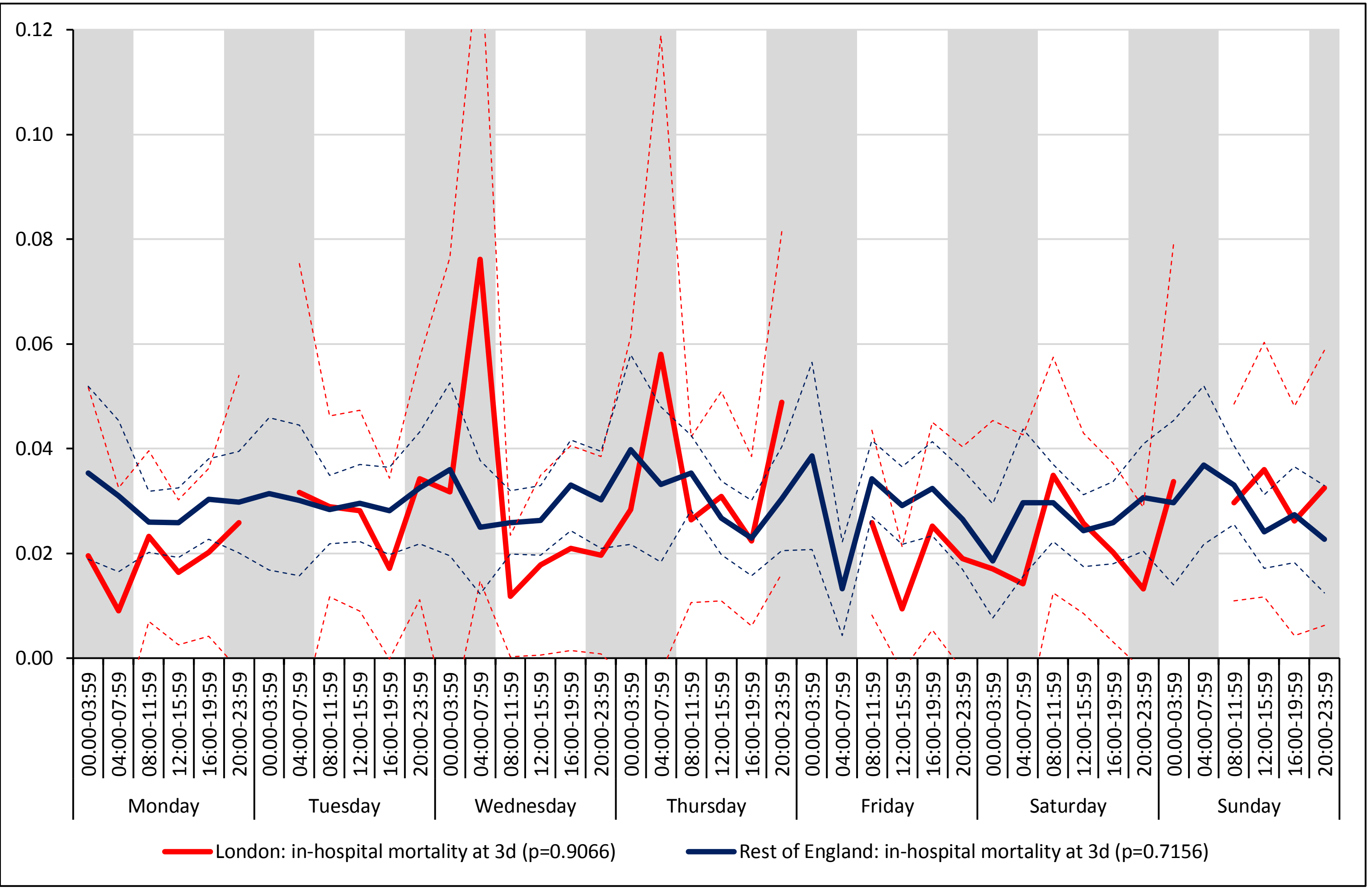

Figure S5(a). Mortality at three days

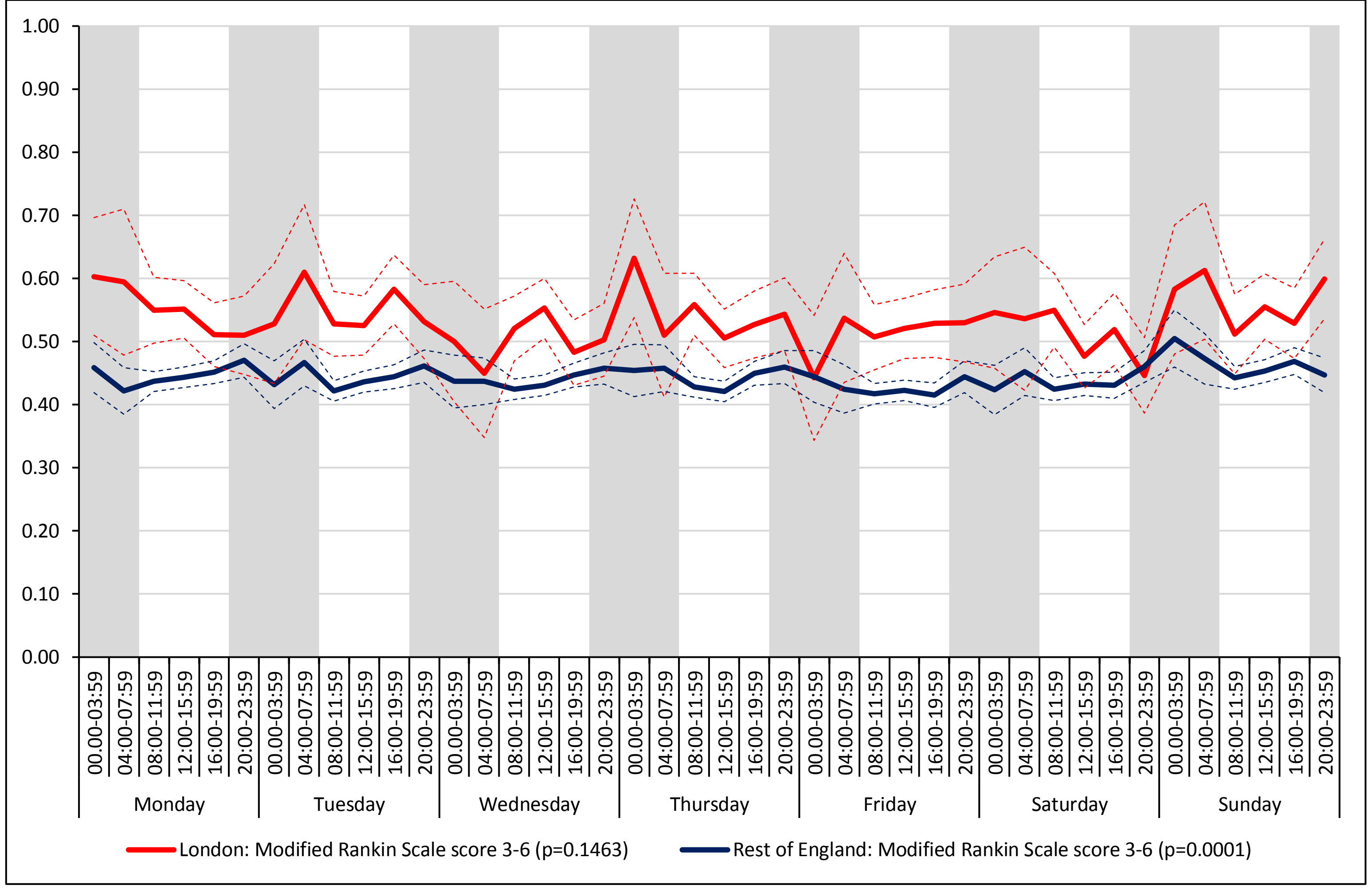

Figure S5(b). Modified Rankin Scale score 3-6

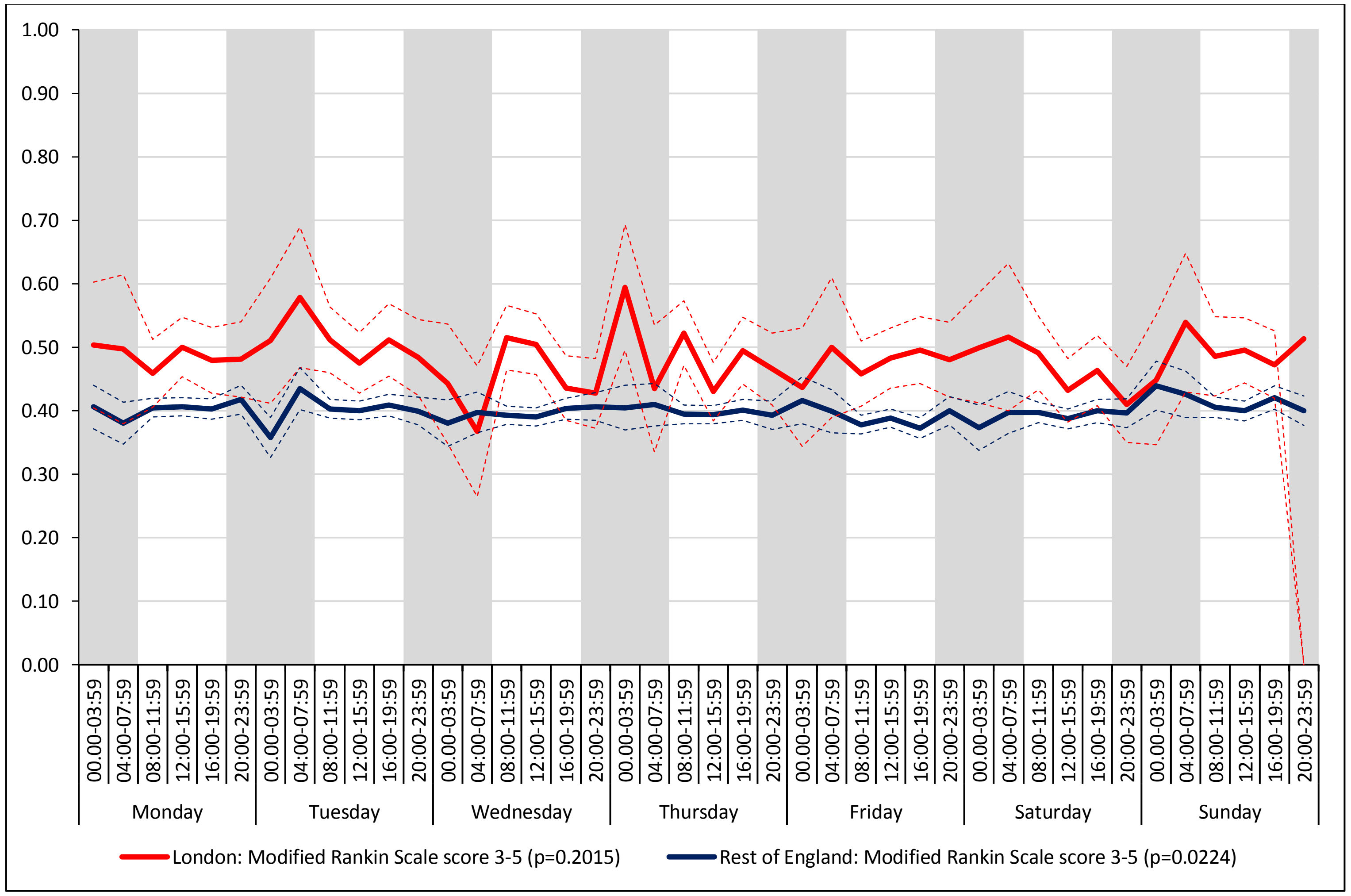

Figure S5(c). Modified Rankin Scale score 3-5

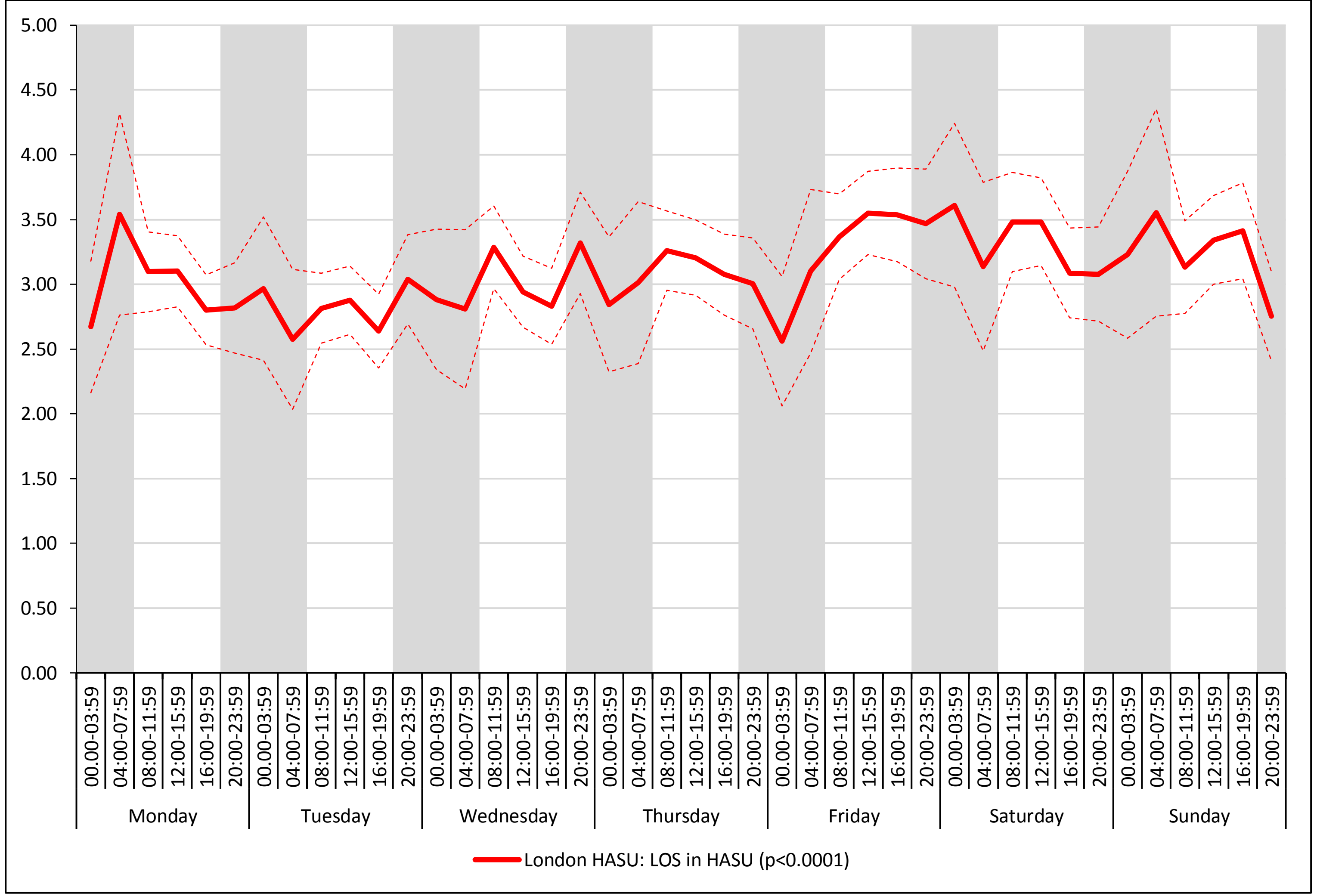

Figure S6(a). Length of stay in HASU

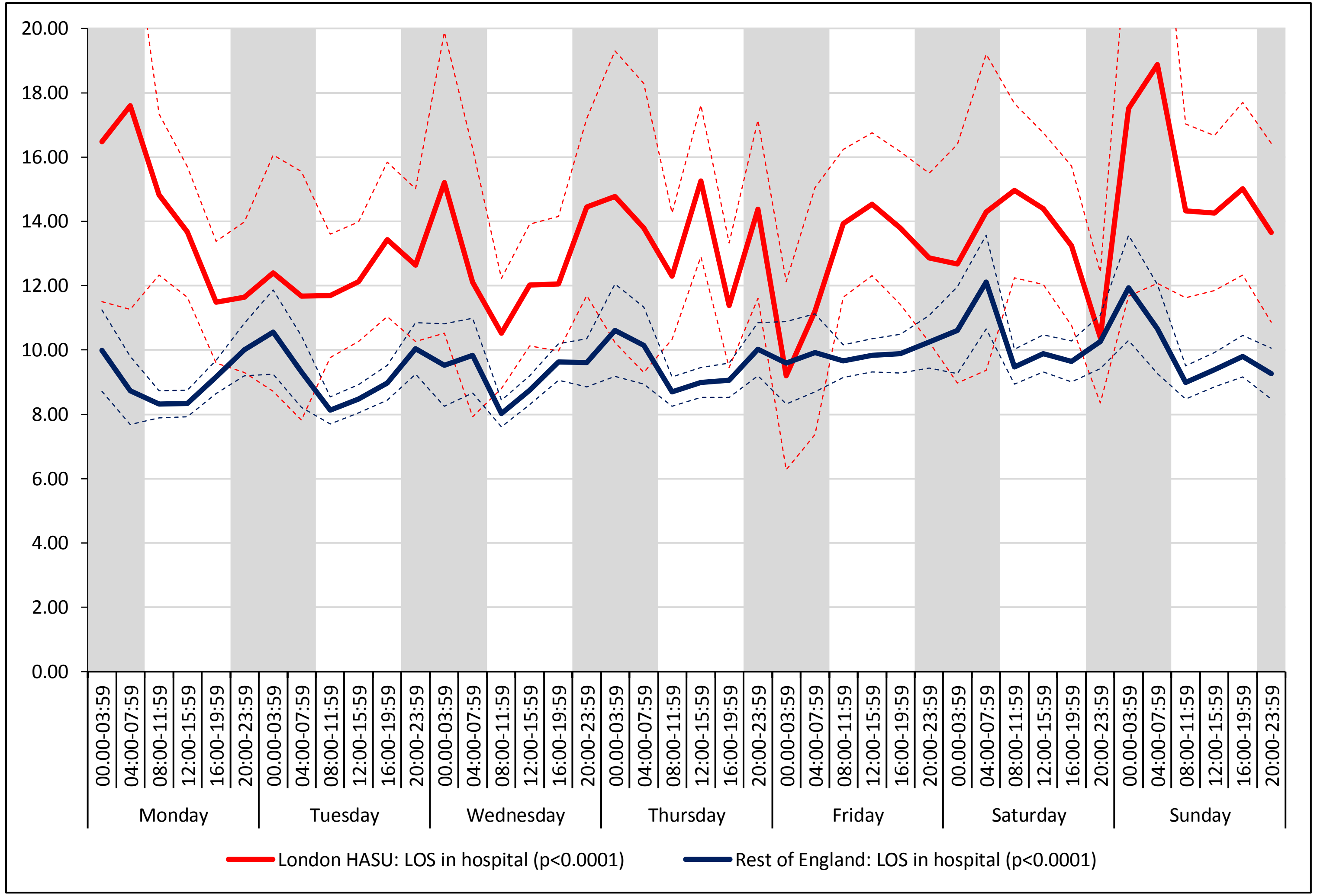

Figure S6(b). Length of stay in hospital
